# Applicability of in vitro mouse lung epithelial cell responses for potency grouping and hazard identification of metal oxide nanoparticles: impact of form, size, surface area, and solubility on toxicity

**DOI:** 10.1007/s00204-025-04285-9

**Published:** 2026-01-28

**Authors:** Andrey Boyadzhiev, Andrew Williams, Sabina Halappanavar

**Affiliations:** 1 Faculty of Science, University of Ottawa, Ottawa, ON Canada; 2 Environmental Health Science and Research Bureau, Health Canada, Ottawa, ON Canada

**Keywords:** Grouping, Engineered nanomaterials, Metal oxide nanoparticles, Benchmark concentration modelling, Prioritization

## Abstract

Human health risk assessment of engineered nanomaterials (ENM; materials with any dimension or structure between 1 and 100 nm) is challenged by the large number of compounds requiring assessment and the intricate association between physicochemical properties and toxicity. Previously, a number of high-content and high-throughput Novel Approach Methodologies (NAMs) were applied to investigate the impact of dissolution on toxicity induced by metal oxide (MOs) nanoparticles (NPs) in lung epithelial cells. This study evaluated the applicability of the data, including information from cell viability, transcriptomics, and genotoxicity endpoints, to conduct potency grouping and hazard identification for 7 individual MOs and their forms—NPs, dissolved equivalents, and bulk microparticle (MPs) analogues (18 total compounds). Benchmark concentration modeling was performed across a range of benchmark responses (BMRs), followed by hierarchical clustering to facilitate potency grouping and hazard identification. Correlation was assessed between endpoints used for potency grouping, and between endpoints and primary particle size, specific surface area, and solubility in cell culture medium across all BMRs. Instantaneously dissolving zinc oxide (ZnO) NPs presented similar potency to dissolved zinc and ZnO MPs, while aluminum oxide, iron oxide, and titanium dioxide NPs showed low potency. Thus, these particles were considered low priority for further testing. Copper oxide, nickel oxide and manganese dioxide NPs exhibited distinct potency and hazards compared to their dissolved or MPs forms, implying these particles warrant further assessment. Material solubility and the form of MOs were associated with endpoint potency. These results demonstrate the applicability of in vitro NAMs-based potency screening for first-tier assessment of MONP-induced acute toxicity.

**Supplementary Information:**

The online version contains supplementary material available at 10.1007/s00204-025-04285-9.

## Introduction

With an increasing focus on including non-animal testing in regulatory assessments, there is growing attention on designing and developing Novel Approach Methodologies for chemical toxicity evaluation (NAMs; defined as technologies, methods, and approaches that provide meaningful information useful for risk assessment activities while avoiding the use of animals (ECHA 2016; EPA 2018)). NAMs include chemical based assays, in vitro assays, computational *in-silico* approaches, and molecular bioassays, such as omics technologies. However, the process for harmonizing these methodologies with current regulatory frameworks, which are often deeply rooted in traditional animal-based testing, has been slow and complex. Any new test method (animal-based or a NAM) must be assessed for its reliability and relevance for the purpose for which it is used (validation), and accepted by a regulatory agency to fulfill an assessment need (regulatory acceptance), before it can be used to generate data in support of regulatory decisions. In addition, a Test Guideline (TG) for each test or a method has to be established according to the OECD’s Mutual Acceptance of Data for harmonized use by international organizations. Given that multiple NAMs may be required to replace a single animal test, the time and resources required to establish a validated and accepted NAM with an associated TG are substantial, hindering the widespread adoption of NAMs.

In the context of risk assessment of engineered nanomaterials (ENMs, defined as any compound with at least 1 dimension or structure within 1–100 nm (ISO 2023)), NAMs are particularly well suited to address the challenges associated with generating data for a variety of ENM comprising combinations of sizes, shapes and surface properties. However, NAMs for commonly reported ENM-induced toxicity are yet to be established. As a result, ENM are presently assessed on a case-by-case basis, which is not sustainable considering the diversity of ENM currently available on global markets and the rate at which new ENM are synthesized.

Previously, we have used an in vitro mouse lung epithelial cell model (FE1) to investigate the toxicity of metal oxide nanoparticles (MONPs) exhibiting different levels of solubility, in addition to microparticle (MPs) and dissolved metal chloride analogues of each. Specifically, we have assessed viability (through the Trypan Blue assay), genotoxicity (DNA strand break formation using the alkaline comet assay; Micronucleus induction by flow cytometry), and perturbations in gene expression (using microarrays) induced by 7 individual MONPs and their analogous forms. Those being zinc oxide (ZnO), copper oxide (CuO), nickel oxide (NiO), manganese dioxide (MnO_2_), aluminum oxide (Al_2_O_3_), iron oxide (Fe_2_O_3_), and titanium dioxide (TiO_2_) nanoparticles (NPs) and MPs; as well as zinc chloride (ZnCl_2_), nickel chloride (NiCl_2_), aluminum chloride (AlCl_3_), and manganese(II) sulfate (MnSO_4_) dissolved metal analogues. A list of all datasets, endpoints, studies, and associated compounds is presented in Table 1. These studies established that soluble metal oxides are more toxic compared to poorly soluble or insoluble MOs and that in addition to solubility, their nano size and chemical composition play a role in toxicity.

**Table 1:** List of published studies from where the endpoint data was derived and the type of data used in the analyses

Published study	Endpoint data (metric) included in the analysis
Boyadzhiev et al. (2021)	24, 48 h Transcriptomics
Boyadzhiev et al. (2023)	24, 48 h Viability (viable cells /cm^2^) 24, 48 h Transcriptomics
Boyadzhiev et al. (2022)	2, 4 h Comet (% DNA in Tail)
Solorio-Rodriguez et al. (2024)	2, 4 h Comet (% DNA in Tail) 40 h Micronucleus (% micronuclei)
Christ (2024)	24, 48 h Viability (viable cells /cm^2^) 24, 48 h Transcriptomics

NPs, nanoparticles; MPs, microparticles

In the present study, the previously published in vitro data (Table 1) was reanalysed to assess its suitability to perform ENM grouping based on their potency and to identify high-risk MONPs for further assessment using advanced toxicity models. In all, data from 7 individual endpoints corresponding to 18 compounds was used in the analysis. In addition, the relationship between the different endpoints, and between endpoints and primary particle size (PPS), specific surface area (SSA), and solubility in cell culture medium were investigated. The results highlight the opportunities and challenges associated with using the existing non-standard in vitro data for informing regulatory decision making.

## Materials and methods

The methodology utilized in this paper is briefly presented below, with additional details available in Online Resource 1.

### Compounds

In total, cellular responses to 18 compounds from 4 recently published manuscripts and a thesis were included in the analyses. Information pertaining to the physicochemical properties of the metal oxide particles as well as supplier information can be found in Table 2.

**Table 2:** Physicochemical properties and supplier information for the 18 compounds

Form	Compound	Supplier (Catalogue Number)	PPS Average (LxW; nm)^a^	Solubility 100 µg/mL (%)^b^	SSA (m^2^/g)^c^
Dissolved	ZnCl_2_	Sigma Aldrich (Z0152-100G)	N/A	N/A	N/A
	MnSO_4_ · H_2_O	Sigma Aldrich (M7899)	N/A	N/A	N/A
	NiCl_2_ · 6H_2_O	Sigma Aldrich (N6136-100G)	N/A	N/A	N/A
	AlCl_3_ · 6H_2_O	Sigma Aldrich (A0718-500G)	N/A	N/A	N/A
Nanoparticle	ZnO NP	US Research Nanomaterials Inc (US3580)	21.65	19.30	27.27
	CuO NP	Sigma Aldrich (544,868)	55.35	51.60	10.34
	MnO_2_ NP	Sky Spring Nanomaterials Inc (4910DX)	24.59	3.87	42.16
	NiO NP	US Research Nanomaterials Inc (US3352)	24.57	1.81	36.60
	Al_2_O_3_ NP	Sigma Aldrich (544,833)	17.30	0.73	145.29
	Fe_2_O_3_ NP	US Research Nanomaterials Inc (US3160)	22.04	N/A	44.88
	TiO_2_ NP	NIST (1898)	23.80	0.05	52.73
Microparticle	ZnO MP	US Research Nanomaterials Inc (US1003M)	1000.00	11.80	5.85
	CuO MP	US Research Nanomaterials Inc (US1140M)	5000.00	1.17	0.80
	MnO_2_ MP	Sky Spring Nanomaterials Inc (4930DX)	5000.00	1.37	2.55
	NiO MP	US Research Nanomaterials Inc (US1014M)	5000.00	0.07	2.52
	Al_2_O_3_ MP	Sky Spring Nanomaterials Inc (1331DL)	950.00	0.02	22.32
	Fe_2_O_3_ MP	US Research Nanomaterials Inc (US1139M)	5000.00	N/A	9.52
	TiO_2_ MP	US Research Nanomaterials Inc (US1017M)	1500.00	0.02	10.76

NP, nanoparticles; MP, microparticles; PPS, primary particle size; SSA, specific surface area; LxW, length x width; N/A, not applicable or not available

^a^Average of length and width. Data from microparticles is manufacturer reported. Data for CuO NPs is taken from (Boyadzhiev et al. 2021). Data for ZnO and TiO_2_ NPs is taken from (Boyadzhiev et al. 2022). Data for NiO and Al_2_O_3_ NPs is taken from (Boyadzhiev et al. 2023). Data for MnO_2_ and Fe_2_O_3_ NPs is taken from (Solorio-Rodriguez et al. 2024)

^b^Data for CuO, NiO, and TiO_2_ particles is taken from (Avramescu et al. 2020). Data for ZnO, MnO_2_, Al_2_O_3_, and Fe_2_O_3_ particles is from (Avramescu et al. 2022)

^c^Data is taken from (Solorio-Rodriguez et al. 2024)

### Benchmark concentration modelling of apical endpoint data

The 24 and 48 h viability data (Boyadzhiev et al. 2021, 2023; Christ 2024), in addition to 2 and 4 h DNA strand break (comet) and 40 h micronucleus (MN) response data reported in (Boyadzhiev et al. 2022; Solorio-Rodriguez et al. 2024) were used for benchmark concentration (BMC) modelling.

BMC modelling was conducted in PROAST Online (version 70.1) at a BMR of 5, 10, 25, and 50% relative risk for viability, comet, and MN data. For viability and comet data collected at 2 different post-exposure timepoints, the timepoint was used as a covariate for each compound tested. The best model was chosen as the one with the lowest Akaike Information Criterion (AIC). If two or more models had identical AIC values, the model with the lowest BMC was chosen. All BMC values were normalized to µM of the constituent metal to allow for accurate comparison between different forms.

### BMC modelling of transcriptomic data

Published datasets corresponding to GSE161017 (https://www.ncbi.nlm.nih.gov/geo/query/acc.cgi?acc=GSE161017) and GSE246159 (https://www.ncbi.nlm.nih.gov/geo/query/acc.cgi?acc=GSE246159), as well as transcriptomic data from (Christ 2024) were used for transcriptomic BMC analysis. The log_2_-normalized fluorescence ratios for biological replicates of each sample were imported into BMDExpress3.0 and a Williams Trend test was conducted to identify genes exhibiting a significant concentration-dependent response at 24 and 48 h. All genes which passed the Williams Trend test (*p* < 0.05), and with a fold change of at least 1.5-fold in either direction were used for BMC modelling with ToxicR averaging. The BMR in BMDExpress3.0 were set to 1.021, 1.349, 1.932, and 2.601 standard deviation which reflect increasing magnitudes of response. The best BMC estimate according to the model average was chosen for each gene.

From the resulting differentially expressed genes (DEGs) and the associated pathways, the ‘HIF1α Signaling’ pathway was identified as the most commonly perturbed pathway across the various compounds investigated and thus, was prioritised for the analysis (Boyadzhiev et al. 2023). BMC estimates associated with 198 genes (corresponded to the IPA content version 101,138,820 (Release Date: 2023-08-24)) belonging to ‘HIF1α Signaling’ pathway, were used. Individual names of genes that make up the ‘HIF1α Signaling’ pathway and those showing concentration-dependent response for each compound is available in Online Resource 2. According to criteria set out by the National Toxicology Program for pathway level BMC analysis, at least 3 genes and 5% of genes within a pathway must have a concentration response to calculate a pathway-level transcriptional point-of-departure (tPOD) (NTP 2018). The 5% cutoff ensures that enough perturbation is present within the pathway to consider it biologically altered. This corresponds to a 10 gene minimum with regards to the ‘HIF1α Signaling’ pathway. In order to retain as much BMC information as possible for clustering, only a 3 gene requirement was used in this manuscript. The median BMC across all concentration-responding genes in the pathway per sample was used as the tPOD for that sample.

### BMC filtering and the final matrices used for downstream analysis

A detailed description of filtering and the composition of the final BMC matrices can be found in Online Resource 1. The final matrices used for downstream analyses are available in Supplementary Tables 1–4 in Online Resource 2.

### Potency grouping using hierarchical clustering with multiscale bootstrap resampling

Hierarchical clustering with multiscale bootstrapping was employed to identify statistically supported clusters for each of the four BMR matrices within resulting dendrograms. For this, the ‘pvclust’ package in R was used, using an nboot = 1000 and iterating the relative sample size of the bootstrap replications between 0.4 and 1.4 × the original dataset size (along 0.1 × increments). An approximately unbiased (AU) *p*-value > 0.95 was used to identify statistically supported clusters. To evaluate the sensitivity of clustering outcomes to different algorithmic assumptions, hierarchical clustering was performed using 12 combinations of linkage (Complete, Average, Single, Ward.D2) and distance metrics (Euclidean, Manhattan, Minkowski). This approach acknowledges that different methods emphasize different aspects of the data structure and allows for assessment of cluster stability across varying configurations. Clusters that consistently appeared across multiple configurations were considered more robust.

The best clustering attempt for each BMR was defined as the linkage-distance combination that resulted in the largest number of statistically supported clusters (AU *p* > 0.95). If multiple solutions resulted in the same number of supported clusters, the solution with the fewest number of singletons (defined as compounds not grouped within supported clusters) was selected as the best.

The presence of compounds in statistically supported clusters (AU *p* > 0.95) was tabulated across all clustering attempts. Compounds not included in any supported cluster in a given attempt were treated as singletons. A binary cluster inclusion matrix was constructed, indicating whether each compound pair co-occurred in a supported cluster for each attempt. This matrix was used to calculate pairwise Jaccard similarity values between compounds, reflecting the proportion of clustering attempts in which two compounds were grouped together. A similarity of 1 indicates that a pair always appeared in the same supported cluster, while 0 indicates they were never grouped together.

### Principal component analysis

Each filtered, winsorized, log_10_ transformed BMC matrix was used to conduct a principal component analysis (PCA) in R using the ‘prcomp’ function (scale = TRUE). PCA is a commonly used dimensionality reduction procedure that can be used to more easily visualize high-dimensionality data while retaining important variance structure. In all cases, 2 principle components (PCs) were kept, representing 71, 77, 90, and 91% total variance at BMR 5, 10, 25 and 50% respectively.

Endpoints were considered to significantly load onto PCs if the |loadings|> 0.4 (Stevens 2009). PCA biplots were created using the ‘factoextra’ plugin and the ‘fviz_pca_biplot’ function. Groups of compounds were highlighted on the biplots based on the groups identified through 12 hierarchical clustering attempts at each BMR (see Sect. ''Potency grouping using hierarchical clustering with multiscale bootstrap resampling'').

### Correlation analyses between endpoints and between endpoints and physicochemical properties

The relationship between each of the 7 endpoints used for potency grouping were assessed through a Spearman’s correlation analysis in R. For this purpose, BMC data across all BMRs was combined into one matrix and used as the input (n = 72). A correlation was considered significant if the *p* < 0.05. An association was considered negligible if |r|< 0.3, weak if 0.31 <|r|< 0.5, moderate if 0.51 <|r|< 0.7, strong if 0.71 <|r|< 0.9, and very strong if |r|> 0.91 (Mukaka 2012). In order to determine relationships between endpoints and key physicochemical properties, the Kendall’s Tau-b was used. An association was considered significant if the *p* < 0.05. For simplicity, the strength of association was judged the same as for the Spearman’s coefficient.

## Results

### Potency grouping at each BMR

In the hierarchical clustering and the resulting classification, a *cluster* refers to a statistically supported cluster formed during hierarchical clustering that includes two or more compounds (AU *p* > 0.95). A *group* is defined as a set of two or more compounds that are likely to be found together within a statistically supported cluster across multiple rounds of hierarchical clustering. These groups represent compounds showing robust associations based on potency differences at each BMR across the 7 endpoints used to define hazard. Compounds which group together across multiple BMRs are expected to have highly similar endpoint response, which can be reliably binned together based on potential hazard. The analysis of all 12 clustering attempts at each BMR revealed 3–4 groups (Groups A-D) (Online Resource 2; Supplementary Fig. 1). Groups ‘A’, ‘C’, and ‘D’ showed variation across BMR while Group ‘B’ did not.

Group ‘A’ consisted of all Zn compounds at BMR5–25, which co-clustered within statistically supported clusters in 67–100% of clustering attempts. At BMR50, ZnO NPs and ZnCl_2_ co-clustered in 83% of attempts, while ZnO MPs only co-clustered with other Zn compounds in 50% of attempts suggesting reduced association at higher effect levels.

Group ‘B’, which contained MnSO_4_ and NiCl_2_, was the most consistent across BMRs co-clustering in statistically supported clusters in 58–100% of clustering attempts. Both dissolved equivalents showed no association with any other compound, except at BMR5, where they co-clustered with ZnCl_2_, ZnO NPs, ZnO MPs, and AlCl_3_ in 17% of clustering attempts.

Group ‘C’, made up of CuO MPs, MnO_2_ MPs, and Fe_2_O_3_ NPs, was observed at BMR5 and through BMR50, with additional compounds co-clustering across clustering attempts as the BMR increases. At BMR25 and 50, Al_2_O_3_ NPs & MPs, Fe_2_O_3_ NPs & MPs, TiO_2_ NPs & MPs, MnO_2_ MPs, and CuO MPs showed a high likelihood of co-clustering. At BMR50, subgroupings in Group ‘C’ were observed consisting of TiO_2_ NPs & MPs, Al_2_O_3_ NPs & MPs and Fe_2_O_3_ NPs co-clustering together in 75–92% of clustering attempts, and another subgroup comprised of CuO MPs and Fe_2_O_3_ MPs which co-clustered in 100% of attempts.

Group ‘D’ was only prominent at BMR5 and BMR50. At BMR5, this group comprised of Al_2_O_3_ NPs and TiO_2_ MPs which appeared in the same statistically supported cluster in 67% of attempts. At BMR50, Group ‘D’ consisted of NiO NPs and MPs which co-clustered in 100% of clustering attempts.

**Fig. 1:**
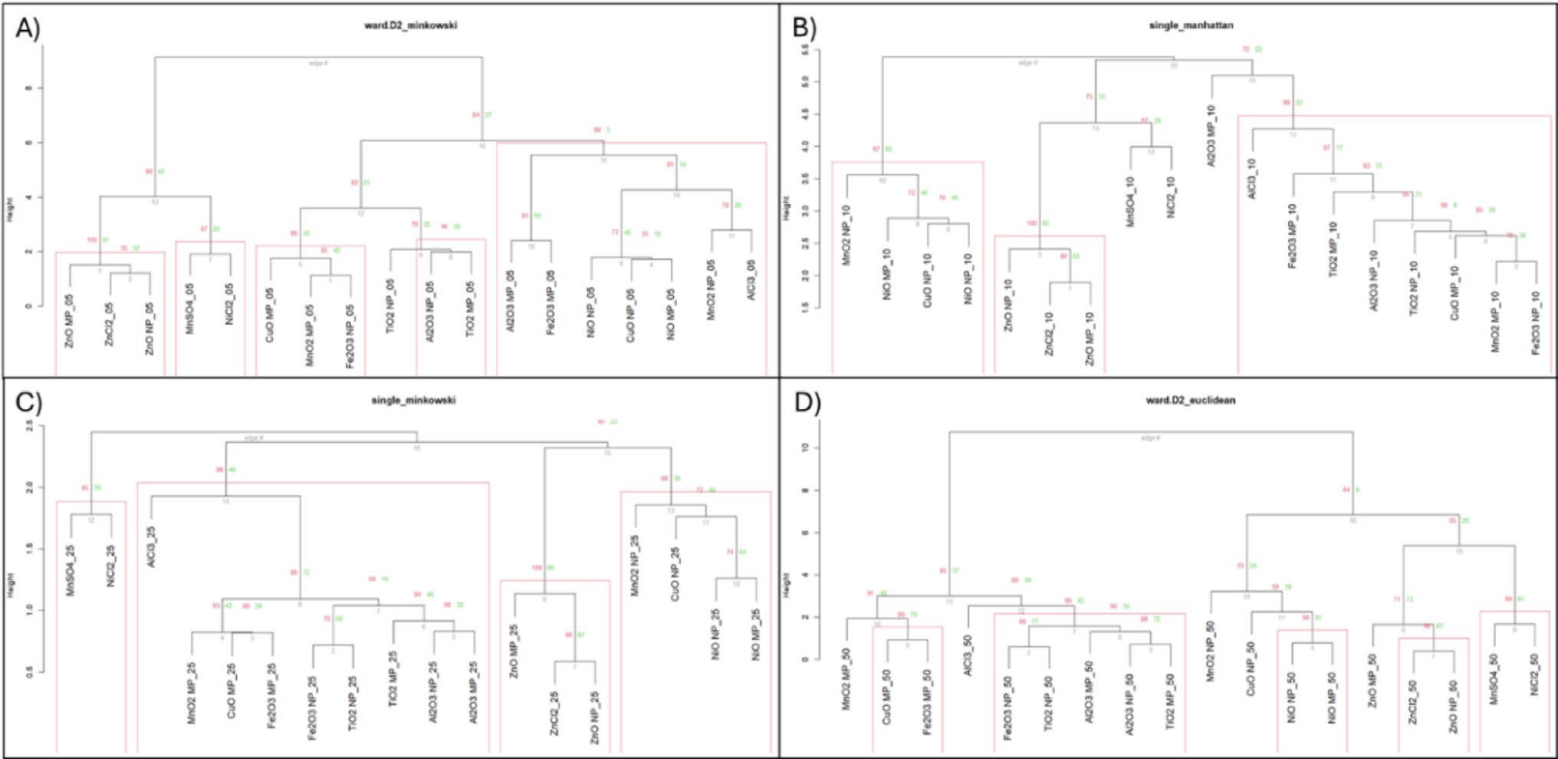
Hierarchical clustering of **A** BMR5, **B** BMR10, **C** BMR25, and **D** BMR50 endpoint matrices, with the best clustering solution depicted. Red boxes: Statistically supported clusters based on 1000 bootstraps with an approximately unbiased *p* > 0.95. Grey numbers: edge number. Green numbers: Bootstrap probability. Red numbers: approximately unbiased *p*-value

Interestingly, CuO NPs and MnO_2_ NPs did not consistently or frequently appear in the same significant cluster with any compound at any BMR (Online Resource 2; Supplementary Fig. 1). Nevertheless, if the best clustering solutions at each BMR are considered, it can be seen that CuO NPs and MnO_2_ NPs frequently appear on the same branch as NiO NPs and MPs, but seldom in a significant manner when all clustering solutions are considered (Fig. 1).

### Visualization of groupings through PCA analysis

Based on the loading and variance analysis at each BMR (Online Resource 2; Supplementary Table 5), PC1 and PC2 explained between 71–91% of the variance in the system, which increases with increasing BMR. In all instances PC1 represents potency to induce decreases in viable cell density and altered signaling through the ‘HIF1α Signaling’ pathway at both 24 and 48 h, with these endpoints loading positively and significantly across all BMRs. Conversely, PC2 represented genotoxicity with both MN induction at 40 h and comet induction at 2 – 4 h loading significantly and in the same direction across all BMRs, except BMR25. At BMR25 MN induction loading was in the same direction as loadings for comet endpoints, but non-significant (loading onto PC2 = 0.38, under the 0.4 cutoff for significance). As the BMR increased, MN and comet endpoints loaded more strongly and in the same direction onto PC1 as viability and ‘HIF1α Signaling’ endpoints. These results across BMRs reveal that separation along PC1 is indicative of potency to induce cellular stress and cell death, and separation along PC2 is a general indicator of genotoxic potential. At higher BMRs (BMR25–50), PC1 partially represented genotoxicity.

**Fig. 2:**
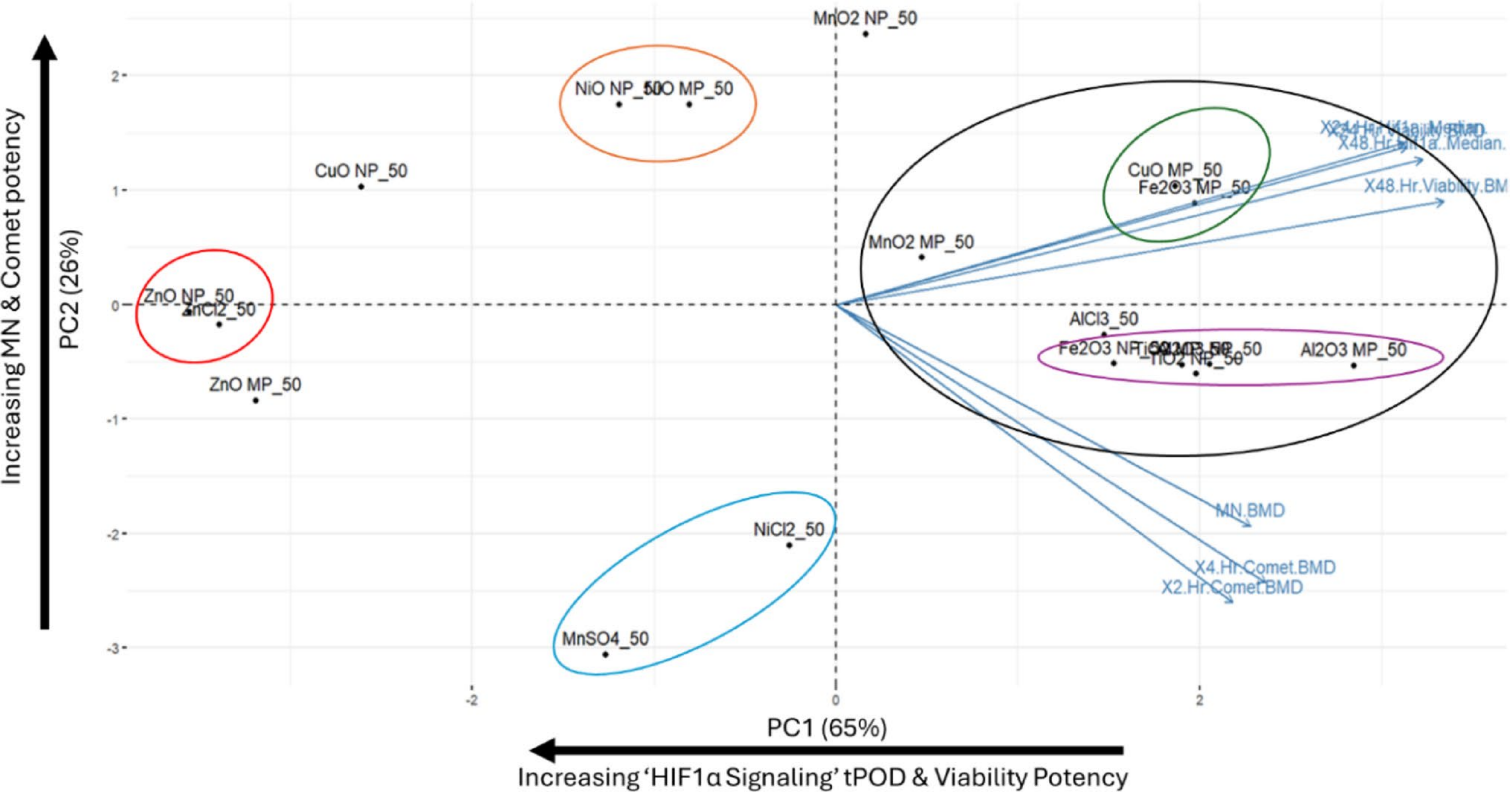
Biplot showing principle component (PC) 1 and PC2 from principle component analysis of the filtered, log transformed, and scaled BMR50 BMC matrix. Vectors indicate the direction of loading for each endpoint. Viability and ‘HIF1α Signaling’ endpoints significantly and positively load onto PC1. MN induction and comet endpoints load significantly and negatively onto PC2. Numbers in parentheses indicate the amount of variance explained by each PC. Colored circles highlight compounds likely to cluster together based on 12 hierarchical clustering attempts at BMR50

When the groups identified at each BMR are used to annotate the PCA biplots, it can be seen that as BMR increases, the compounds become more separated and groupings identified through hierarchical clustering become more apparent, with BMR50 resulting in the greatest separation and delineation of groups (Fig. 2. Online Resource 2; Supplementary Fig. 2–4). Detailed descriptions of group placement along PC1 and PC2 across BMRs are provided in Online Resource 4.

### Potency grouping across BMR5-50 and overall compound grouping

**Fig. 3:**
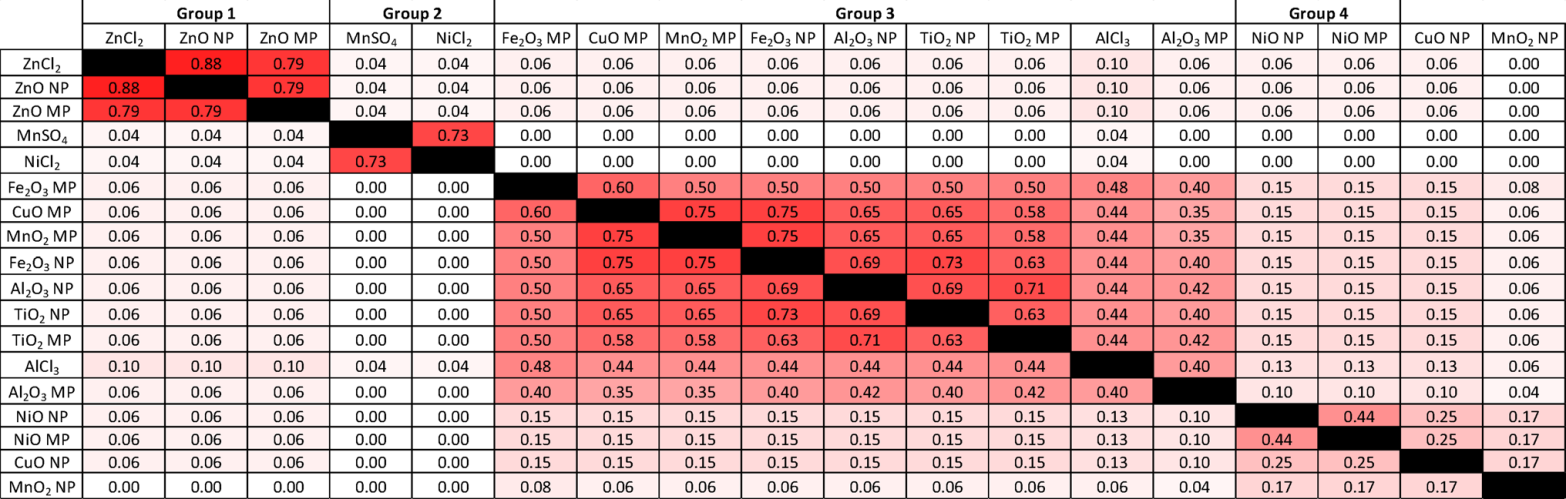
Likelihood of MONPs, MPs, and dissolved metals to co-cluster based on potency to induce 7 in vitro endpoints across 48 clustering attempts from BMR5-50. 1: compounds always appear in the same statistically supported cluster. 0: compounds never appear in the same statistically supported cluster. Red: higher likelihood of appearing in the same statistically supported cluster. Black: non-applicable comparison. 1–4: Potency groups based on likelihood to appear in the same statistically supported cluster across BMR5–50

When significant clusters across all 48 attempts from BMR5–50 are tabulated and a Jaccard similarity measure is conducted on the resulting matrix, 4 main groups emerged (Fig. 3). Group ‘1’ included all Zn substances, with ZnO NPs and ZnCl_2_ slightly more likely to cluster together than with ZnO MPs. This group appears at all BMRs. Group ‘2’ consisted of dissolved MnSO_4_ and NiCl_2_, which also appears at all BMRs. Group ‘3’ contained poorly soluble–insoluble Al_2_O_3_ NPs & MPs, TiO_2_ NPs & MPs, Fe_2_O_3_ NPs & MPs, CuO MPs, and MnO_2_ MPs. Group ‘3’ appears at all BMRs, however additional compounds become part of this group as BMR increases. Finally, Group ‘4’ included NiO NPs and MPs, which only became prominent at BMR50.

### Endpoint-endpoint and endpoint-property correlation analyses

**Fig. 4:**
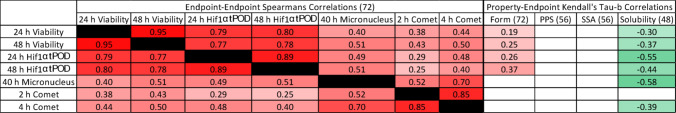
Significant Spearman’s correlations between endpoints, and Kendall’s Tau-b correlations between endpoints and specific surface area (SSA), primary particle size (PPS), and % solubility. Blank cells designate a non-significant association or non-applicable associations. An association was considered significant if the *p* < 0.05. Green: significant negative association. Red: Significant positive association

As a final step, the Spearman’s rank test and the Kendall’s tau-b correlation test were utilized to assess the associations between endpoints used for potency grouping and hazard identification and between endpoints and SSA, PPS, and solubility in cell culture medium respectively across all BMRs (Fig. 4).

All endpoints were positively correlated with each other. Viability and ‘HIF1α Signaling’ endpoints were strongly to very strongly correlated with each other (correlation coefficient between 0.77 and 0.95). All genotoxicity endpoints (MN formation and 2 & 4 h comet assay) showed weak to moderate correlations with viability and perturbation of the ‘HIF1α Signaling’ pathway (correlation coefficient between 0.25 and 0.51). In contrast, MN induction and 4-h comet assay results were more strongly correlated with each other (correlation coefficient = 0.70), while 2 and 4 h comet assay results were strongly correlated (correlation coefficient = 0.85).

With respect to physicochemical properties, PPS and SSA showed no association with the potency of compounds to induce endpoint response. The property called ‘form’, which is an ordinal characteristic that describes whether a compound is a dissolved metal (1), an NP (2), or an MP (3), showed significant positive, but negligible associations with viability and perturbation of ‘HIF1α Signaling’ at 48 h. The association between ‘form’ is weak with respect to 48 h ‘HIF1α Signaling’ potency. These associations indicate that MONPs and MPs are less potent in inducing reductions in viable cell density or perturbing ‘HIF1α Signaling’ as compared to fully dissolved metals when the concentration metric is normalized to the amount of constituent metal in the exposure medium.

Finally, solubility at 100 µg/mL is the property most strongly associated with endpoint potency, showing weak to moderate significant negative associations between BMC in all endpoints except for 2 h comet. This indicates that as particle solubility in the extracellular medium increases, overall potency to induce endpoint response increases.

### Hazard identification per group

The endpoints used for potency grouping can be used as indications of potential hazard. Reduction in viable cell density is indicative of cell death or cytotoxicity. Perturbation of the ‘HIF1α Signaling’ pathway can be interpreted as cellular stress. Response through the comet assay indicate DNA strand break formation (type of genotoxic hazard), and finally MN response is indicative of clastogenic/aneugenic potential.

Based on Spearman’s correlations of endpoint-endpoint potency it was observed that different timepoints for the same endpoint are strongly correlated. In order to make a positive or negative call for hazard identification, a cutoff was set. The maximal response for each endpoint at the latest timepoint of assessment was used. In terms of cell death, a cutoff of ≤ 66% viable cells remaining at the highest concentration (1.5-fold decrease in response over control) was used. For clastogenicity/aneugenicity assessed through MN induction, a cutoff of ≥ 2-fold response at concentrations exhibiting ≥ 40% viability. For DNA strand break induction measured using the comet assay, the cut-off was set at > 10% DNA in tail. Finally, for cell stress signaling assessed via the ‘HIF1α Signaling’ pathway, a positive response was defined as the presence of at least three concentration-responsive, differentially expressed genes within a given sample. (Table 3).

**Table 3:** Hazards identified for each potency grouping determined through BMR5–50 clustering. Check mark indicates that compound is positive for that hazard category

Group	Compound	Hazard			
		Cell Death	Cell Stress Signaling	DNA Strand Break Induction	Clastogenicity/Aneugenicity
1	ZnCl_2_	✓	✓	✓	✓
	ZnO NP	✓	✓	✓	✓
	ZnO MP	✓	✓	✓	✓
2	MnSO_4_	✓	✓		
	NiCl_2_	✓	✓		
3	MnO_2_ MP		✓	✓	✓
	CuO MP				✓
	Al_2_O_3_ NP		✓		✓
	AlCl_3_		✓		✓
	Al_2_O_3_ MP				
	Fe_2_O_3_ NP		✓		
	Fe_2_O_3_ MP		✓		
	TiO_2_ NP	✓	✓		
	TiO_2_ MP				
4	NiO NP	✓	✓	✓	✓
	NiO MP	✓	✓	✓	✓
	CuO NP	✓	✓	✓	✓
	MnO_2_ NP		✓	✓	✓

Group ‘1’ containing Zn compounds was positive for all hazards identified, with all Zn compounds exhibiting the highest potency to induce cell death and cell stress signaling. Group ‘2’ containing dissolved NiCl_2_ and MnSO_4_ was positive for cell death and cell stress signaling hazards, but was negative for both types of genotoxicity. Group ‘4’ containing NiO NPs and MPs was positive for all hazards, with moderate potency to induce cell death and cell stress signaling, but the highest overall potency to induce DNA strand breaks. Group ‘3’ containing 9 insoluble MONPs, MPs, and AlCl_3_ was almost entirely negative for cell death and DNA strand break induction. However 6/9 compounds were positive for induction of cell stress signaling, although this drops to 2/9 if only the 24 h timepoint was considered (Online Resource 2; Supplementary Table 1–4). In Group ‘3’, 4/9 samples were positively identified for clastogenicity and aneugenicity based on a twofold increase in MN frequency without a reduction in viability < 40%. Of these samples, AlCl_3_ presented a similar potency to induce clastogenicity / aneugenicity as compared to CuO NPs and NiO NPs at BMR50 while the rest of the samples presented moderate to low relative potency (Online Resource 2; Supplementary Table 1–4).

## Discussion

In this study, the application of in vitro concentration–response data for potency and hazard grouping of ENM was investigated using BMC potency estimates and hierarchical clustering approaches. Correlations between the individual endpoints, and between endpoints and ENM physicochemical properties were determined. The results were used to identify high-risk ENM for further assessment using complex models. Through this exercise, several key observations were made regarding the impact of form (dissolved, MONP, MP) on toxicity, which can be used as weight-of-evidence to support ENM grouping for risk assessment. The implications and limitations of the current in vitro data for ENM risk assessment activities, and directions for future work are discussed.

### Endpoint potency is associated with form and particle solubility

Primary particle size, specific surface area, and solubility are all properties considered important to the toxicity of MONPs. Based on BMC estimates across BMR5–50, both form and solubility were significantly associated with endpoint potency (Fig. 4). Viability and cellular stress (altered ‘HIF1α Signaling’ pathway) were positively influenced by these properties, while the genotoxicity endpoints were only influenced by solubility. Dissolved equivalents showed higher potency in inducing cell death and cellular stress signaling, followed by MONPs and then MPs. Solubility was negatively correlated with response through all endpoints, except comet induction at 2 h, implying that as particle solubility increases, toxic potency increases. This aligns with the general mode-of-action of metal oxides, in which soluble compounds can release metal ions into the surrounding microenvironment, potentiating a rapid physiological response. More importantly, for ENM investigated in this study, the nano form showed higher potency compared to bulk analogues, warranting further assessment of MONPs.

### Potency grouping & hazard identification

ZnO NPs and Al_2_O_3_ NPs showed a tendency to induce similar hazard in vitro based on endpoint potency data derived from different forms of metal oxides (Table 3).

Potency of instantaneously dissolving ZnO NPs across endpoints was similar to the potency of dissolved Zn ions. Zn compounds showed a high likelihood to group together across all BMRs. The human health risk assessment of ZnO conducted by the EU has identified the zinc cation as the determining factor for toxicity, with the caveat that the physicochemical properties of ZnO will affect toxicokinetics and bioavailability, and therefore may have an impact on resulting toxicity (ECHA 2016). In a more recent draft screening assessment completed by Health Canada and Environment and Climate Change Canada, ZnO was grouped alongside 75 zinc compounds for evaluation (ECCC & HC 2019). The report did not specify hazards related to zinc inhalation other than the well characterized systemic immune response known as metal fume fever. It is important to note that this evaluation specifically excluded nano forms of Zn or ZnO. In human volunteers exposed to ZnO NPs and MPs via inhalation for 2 h, similar effects were observed between the two forms with respect to induction of inflammatory markers in the blood, though slightly stronger effects were seen with MPs as compared to NPs (Monsé et al. 2021). Based on potency grouping (similar endpoint potency, Fig. 3) and hazards identified (similar hazard, Table 3), including induction of similar transcriptional pathways for all three forms (Boyadzhiev et al. 2023), the in vitro data generated provides support for read-across of dissolved Zn toxicity to that of pristine ZnO NPs. Thus, a separate evaluation of ZnO NPs may not be warranted, which is in alignment with the instantaneously dissolving ENM predefined grouping hypothesis recommended by the Grouping, Read-Across, Characterisation and classification framework for regulatory risk assessment of manufactured nanomaterials and Safer design of nanoenabled products (GRACIOUS) (Braakhuis et al. 2021). However, it is important to note that ZnO solubility is dependent on the pH of the medium and in microenvironments where ZnO solubility is reduced, the potency of ZnO NPs may differ. This emphasizes the importance of solubility testing in a range of media representative of a broad spectrum of physiological and natural environments.

Al_2_O_3_ NPs which present between 0.73 and 1.11% dissolution in the cell culture medium used in the present study (Avramescu et al. 2022) showed similar potency to Al_2_O_3_ MPs and AlCl_3_ to induce loss in viable cell density, and DNA damage responses at BMR50, but varied potency from BMR5–25. Since BMR50 is representative of what is considered a biologically significant in vitro response (1.5-fold increase), similarity in potency at this BMR was weighed more strongly than at lower BMRs. However, the ability to alter both ‘HIF1α Signaling’ and induce MN differed across all BMRs, with AlCl_3_ > Al_2_O_3_ NPs > Al_2_O_3_ MPs (Online Supplement 2; Supplementary Table 1–4). The dissolved AlCl_3_ forms insoluble precipitates in cell culture medium (Boyadzhiev et al. 2023; Helmig et al. 2018) and therefore, Al_2_O_3_ might show dichotomous behaviour, exhibiting properties of both particle and dissolved ions. The human health risk assessment of aluminum compounds makes note of the unstable nature of Al^3+^, with its preference to bind to carboxylate and phosphate groups, in addition to tyrosine residues (Krewski et al. 2007). To date, no direct comparisons between the solubility of Al_2_O_3_ NPs and MPs have been made in artificial lysosomal fluid (ALF), but it is known that Al_2_O_3_ NPs have a capacity to dissolve in ALF (Holmfred et al. 2022) and it is known that at the pH of lysosomes (4.5–5.0), Al^3+^ is the predominant form of dissolved Al present (Krewski et al. 2007). AlCl_3_ was shown to have the highest potential of the three forms to induce MN (Solorio-Rodriguez et al. 2024), and AlCl_3_ has been shown to bind the phosphate groups in the DNA backbone and on the guanine site of G-C base pairs through chelation (Ahmad et al. 1996). Furthermore, AlCl_3_ can lead to the cross-linking of chromosomes in Novikoff ascites hepatoma cells (Wedrychowski et al. 1986), and chromosomal instability and clastogenicity in mammary epithelial cells (Mandriota et al. 2020). Despite these reports, aluminum compounds have not been shown to be carcinogenic in animal models (Krewski et al. 2007). Thus, MN induction after exposure to Al_2_O_3_ NPs could be the result of intracellular particle dissolution, releasing Al^3+^ ions which associate with the DNA in the nucleus or during mitosis, causing chromosomal cross-linking, and subsequent MN formation. Increased potency of AlCl_3_ to induce perturbations in ‘HIF1α Signaling’ as compared to Al_2_O_3_ NPs and MPs could be due to the presence of dissolved Al in the cell, which induced a subtle concentration-dependent increase in the expression of 3–5 genes in AlCl_3_ or Al_2_O_3_ NPs exposed cells at 48 h, but not Al_2_O_3_ MPs (Online Resource 3). Overall, these results suggest that Al_2_O_3_ NPs are not expected to induce toxicity in the lung following inhalation and do not warrant further testing.

### Potency grouping & hazard identification: MONPs with hazard akin to bulk forms

In total, there were 3 MONPs which showed the same in vitro hazard as their bulk forms (Table 3). These are poorly soluble–insoluble NiO NPs and MPs, which group together in Group ‘4’ and are positive for all tested hazards; insoluble Fe_2_O_3_ NPs & MPs which group together in Group ‘3’ and are positive for cell stress signaling; and finally, poorly soluble MnO_2_ NPs and MPs which appear in separate groupings but are positive for cell stress signaling, DNA strand break induction, and MN induction.

Both NiO NPs and MPs presented a moderate potency to induce cell death and cell stress signaling and a strong potency to induce genotoxicity. In contrast, NiCl_2_ was more potent at inducing cell death and cell stress signaling, but much less potent in inducing genotoxicity. A recent tier-II human health screening assessment of NiO conducted by the Australian government showed that NiO merits classification as a category 1 skin sensitizer, pulmonary toxicant through repeated inhalation, and 1A carcinogen according to the GHS classification scheme (NICNAS 2014). The carcinogenic potential of Ni compounds lies in their ability to induce epigenetic silencing and interfere with the functioning of cellular maintenance machinery, including DNA repair enzymes (Genchi et al. 2020; Hartwig et al. 2002). This is mainly believed to be the result of dissolved Ni, which is also the basis for dermal sensitization resulting from chronic skin exposure to Ni compounds. While NiO NPs exhibit poor-negligible solubility in cell culture medium (Avramescu et al. 2020), they dissolve better in ALF compared to the larger size NiO (Shinohara et al. 2017). The NiO MPs investigated in the present study exhibited a mean particle size of 5 µm as reported by the manufacturer, however (SEM) imaging revealed the presence of a nano size fraction of NiO (Boyadzhiev et al. 2023), which may explain the similarities in NiO NPs and NiO MPs potency across the endpoints. Although almost all endpoints were similarly induced by NiO NPs and MPs, the former did present a much lower BMC for 48 h reduction in viable cell density as compared to NiO MPs (Online Resource 2; Supplementary Table 1–4). While both NiO NPs and MPs induce the same acute hazards, enhanced uptake and dissolution of the NPs could manifest in more pronounced pulmonary toxicity in vivo. Thus, the results suggest prioritizing NiO NPs for further testing.

For Fe_2_O_3_ NPs and MPs, a positive call was only seen for cell stress induction, with 3–6 genes showing concentration-dependent response at either timepoint (Online Resource 3). Fe_2_O_3_ NPs presented weak viability and genotoxicity potency, and a subtle ability to induce cell stress signaling through the ‘HIF1α Signaling’ pathway, similar to that observed for Fe_2_O_3_ MPs, especially at the largest BMR of 50% (Supplementary Table 4). However, pronounced differences between the two sizes were apparent when the entire transcriptomic responses were considered; exposure to Fe_2_O_3_ NPs resulted in substantially more DEGs and enriched pathways compared to MPs at a given concentration (Christ 2024). To date, a human health risk assessment has not been conducted for Fe_2_O_3_, however research indicates a pulmonary hazard from inhalation exposure (EFSA 2016). Since Fe_2_O_3_ is a persistent particle with low solubility in both neutral and acidic environments, it can accumulate in the lung after inhalation. This leads to the development of an interstitial lung disease known as siderosis (Akar et al. 2018; Doğrul 2017). With respect to Fe_2_O_3_ NPs, they show limited dissolution in ALF (Cook 2022), and analysis of DEGs induced by Fe_2_O_3_ NPs over a 48 h period shows perturbation of pathways associated with the transport of iron (Christ 2024). Based on this, while Fe_2_O_3_ bulk particles can be considered insoluble and present a persistent particle hazard with low potential for acute pulmonary toxicity, Fe_2_O_3_ NPs may present an additional hazard related to dissolved iron ions in the lung due to dissolution within lysosomes. Thus, the results from the present study may not be sufficient to draw a conclusion regarding prioritisation of Fe_2_O_3_ NPs for further testing.

Both MnO_2_ NPs and MPs induced cell stress signaling and genotoxicity (DNA strand breaks and MN formation) with a weaker ability to induce cell death (Table 3). At BMR50, MnO_2_ NPs were more potent at inducing genotoxicity than MnO_2_ MPs, while the MPs were more potent at inducing cell stress through the ‘HIF1α Signaling’ pathway. However, similar to Fe_2_O_3_ NPs at the transcriptional level, exposure to MnO_2_ NPs resulted in a larger number of DEGs showing concentration-dependent response than MnO_2_ MPs, even though the latter presents lower overall BMCs (Online Resource 2; Supplementary Table 1–4). Manganese is an essential micronutrient, and similar to nickel, can result in stabilization of HIF1α (Li 2006) and the induction of chemical hypoxia in exposed cells and tissues (Han et al. 2009). Cellular hypoxia is associated with oxidative stress, protein folding stress, and changes in metabolism from oxygen intensive processes such as cellular respiration to glycolysis, which result in mitochondrial dysfunction. The main target organ for manganese induced toxicity is the brain, where neurotoxicity manifests as manganism with similarities to Parkinson’s disease (Racette 2014). A human health risk assessment has been conducted for inhalation of manganese, which also included information pertaining to MnO_2_ (Health Canada 2010). Due to the target organ for toxicity being the brain, the assessment mainly focused on neurotoxic effects of manganese exposure. Both MnO_2_ NPs and MPs showed poor solubility in cell culture medium, with NPs presenting higher solubility than MPs at the same concentration (Avramescu et al. 2022) and a fivefold higher release of dissolved Mn in ALF as compared to cell culture medium (Avramescu et al. 2022). Taken together, these results suggest NPs present a greater potency than the MPs, which could be driven by intracellular MnO_2_ dissolution and cellular manganese overload. Overall, in vitro endpoint screening identified the same hazards for both MnO_2_ NPs and MPs, however MnO_2_ NPs should be prioritized for further testing due to their enhanced potency (as determined based on different grouping for both forms) and solubility as compared to the bulk compound, potentially allowing for more intense local effects in the lung and greater systemic absorption of dissolved manganese.

### Potency grouping & hazard identification: MONPs with distinct hazard

There were two MONPs which had distinct hazard calls as compared to their bulk and dissolved counterparts. First is TiO_2_ NPs from Group ‘3’, which presented positive calls for cell death and increased cell stress signaling, which was not observed for their MP analogues. Next is CuO NPs which clustered independently with positive calls in all hazard categories in comparison to CuO MPs in Group ‘3’, which were positive only for clastogenicity / aneugenicity.

The in vitro results from the present study suggest that TiO_2_ NPs present additional hazard compared to MPs, although they both appear in the same potency Group ‘3’ (Table 3). The International Agency for Research on Cancer (IARC) has classified TiO_2_ as a class 2b carcinogen in 2006 (Baan et al. 2006) based on studies showing tumorigenesis in rats following inhalation of TiO_2_. More recently, the food-grade pigment E171 potentially containing TiO_2_ NPs was banned from use in the EU due to concerns relating to immunotoxicity, genotoxicity, and potential neurotoxicity arising from the nano component (Younes et al. 2021). In Canada, a recent draft screening assessment of titanium compounds, which included TiO_2_ but not TiO_2_ NPs, concluded that there was a low risk for human and environmental harm from TiO_2_ and it does not meet the criteria for regulation (ECCC & HC 2023). TiO_2_ NPs did not induce genotoxicity in either the CometChip® or MicroFlow® assays (Boyadzhiev et al. 2022; Solorio-Rodriguez et al. 2024). At BMR50, TiO_2_ NPs and MPs presented similar potency across all endpoints due to highly imprecise estimation of viability potency for TiO_2_ NPs at this BMR (BMC = 66.21–720 µM Ti at BMR5–25, while BMC = 2712–5747 µM Ti at BMR50 for 24 and 48 h viability endpoints; Online Resource 2; Supplementary Table 1–4), At BMR5-25, TiO_2_ NPs consistently presented higher potency to induce reductions in viable cell density and to induce cell stress signaling through the ‘HIF1α Signaling’ pathway (Online Resource 2; Supplementary Table 1–3). However at the higher BMR50, the results could not be differentiated between the NPs and MPs. At the transcriptional level, TiO_2_ NPs induced altered expression of a larger number of DEGs and perturbed associated pathways (Boyadzhiev et al. 2023). TiO_2_ MPs and NPs tended to cluster separately based on canonical pathway enrichment, indicating differences in the types of pathways enriched, and their activation states (Boyadzhiev et al. 2023). While similar potency is observed for TiO_2_ NPs and MPs in most endpoints, including primary genotoxicity, TiO_2_ NPs presented greater loss in viable cell density and cell stress signaling potency, especially at 48 h, which could manifest in greater pro-inflammatory potential in vivo. These results suggest further prioritisation of TiO_2_ NPs may not be required.

The final MONP with distinct in vitro hazard calls from its dissolved and bulk equivalents is CuO NPs. CuO NPs were positive for all hazards, and clustered by themselves away from all other compounds (Table 3.). In contrast, CuO MPs only presented a positive hazard call for clastogenicity / aneugenicity. While CuCl_2_ was not included as part of this grouping and hazard identification exercise, it was shown to induce viability loss and alter cell stress signaling calls but did not induce genotoxicity (Boyadzhiev et al. 2023; Solorio-Rodriguez et al. 2024). Solubility testing in cell culture medium showed pronounced differences between CuO NPs and MPs, with NPs being soluble while MPs were poorly soluble (Boyadzhiev et al. 2021). In ALF, CuO NPs showed high solubility, indicating a rapid ability to dissolve when endocytosed (Chakraborty and Misra 2019). In a 2008 human health risk assessment of copper containing substances, risk evaluators made note of the classification of copper(I) oxide and copper oxychloride as acutely hazardous following inhalation, however CuO (copper(II) oxide) were specifically not included (ECHA 2008). CuO NPs presented a distinct acute hazard compared to CuO MPs in the present study, with much higher solubility potential. Thus CuO NPs merit prioritisation for further testing.

### Limitations and future directions

In this manuscript, we introduce a novel approach to potency grouping that leverages potency estimates across multiple BMRs to derive groupings that more accurately capture differences in relative responses across toxicity endpoints and compounds. A current limitation, however, is the lack of batch-processing capability for BMC modeling in PROAST, which required us to run each endpoint-BMR combination manually. While this was manageable for the relatively small set of compounds and endpoints considered here, applying the method to larger datasets would greatly increase the workload. Implementing batch processing in PROAST would substantially streamline the workflow and facilitate routine, scalable application of this potency-grouping methodology to in vitro NAM data for both ENMs and conventional chemicals.

Currently, validated in vitro NAMs that are relevant to ENM and ready to support regulatory decision making are not available, although there are many ongoing activities globally to develop and adapt regulatory testing guidelines and NAM assays for ENM (Hristozov et al. 2024). However, as demonstrated in this study, in vitro endpoint data can be applied for first-tier screening for organ-specific toxicity, as demonstrated in this study, with some limitations.

While the in vitro data utilized in this study are promising for acute toxicity testing, addressing long-term effects such as carcinogenicity remains a challenge. The genotoxicity assays can only detect primary (direct or indirect) genotoxicity. Persistent MONPs such as TiO_2_ or Fe_2_O_3_ NPs, if deposited in sufficient quantity in lungs or following repeated exposure, can induce chronic tissue inflammation that may result in oxidative stress and secondary genotoxicity, which cannot be accurately captured at the time points assessed with the single cell type used in this work. Thus, assessment of the full spectrum of toxicological hazards addressed by traditional animal tests, particularly those involving multiple organ systems or complex mechanisms is a challenge.

Current regulations often rely heavily on in vivo data, which is not available at present for comparison with in vitro data for all ENMs to demonstrate comparable or superior performance of NAMs. A literature review was conducted to identify in vivo reports involving the various MOs and their respective forms investigated in this study; however, no relevant datasets were found to enable comparison. A paired exposure approach was previously applied to Mitsui-7 carbon nanotubes, involving FE1 cells exposed in submerged conditions similar to the present study and in vivo exposure via intratracheal instillation in C57BL/6 mice (Søs Poulsen et al. 2013). Transcriptomic analysis revealed that both models exhibited perturbations in similar pathways related to inflammation and oxidative stress, although the specific DEGs varied between models. A similar paired exposure to graphene nanoplatelets and single layer graphene was carried out as part of the European Union led PLATOX project (FP7 SIINN ERA-NET program). The results showed that cytotoxicity and DNA strand break induction after 24 h exposure in vitro was predictive of inflammation and DNA strand break formation in vivo following 28 day inhalation, and that in vitro potency ranking matched the in vivo potency (Creutzenberg et al. 2022).

Conducting a comparable in vitro*–*in vivo paired exposure using a subset of the same MONPs in lung epithelial cells, and inhalation exposure in mice would offer valuable insights into the accuracy of the groupings identified in the present study. Additionally, it would provide empirical evidence linking in vitro endpoints with in vivo apical toxicity, while also highlighting potential species-specific differences in cellular signaling responses between mice and humans. However, with a push to replace animal testing in regulatory assessments, it will be next to impossible to conduct such pairwise studies. The challenge then is to validate novel NAMs independent of comparable in vivo data. Alternative strategies that build scientific confidence in these in vitro models are available. For example, use of mechanistic validation, such as demonstrating that NAMs reliably predict critical biological processes and pathways associated with ENM-induced toxicity, including oxidative stress, inflammation, or DNA damage. Combined with systems biology and computational modeling, the connections between the different endpoints assessed in vitro and an adverse outcome in vivo can be strengthened. Inclusion of multiple cell types and assays can enhance robustness and reproducibility, and in the absence of animal data, showing consistency in response across model systems and endpoints can validate the toxicity potential and underlying mechanisms. Lastly, benchmarking NAMs (e.g., transcriptomic signatures) against well-characterized reference ENMs with known human health effects can provide indirect but meaningful validation, ensuring that the models capture relevant biological responses.

## Conclusions

In conclusion, in this study, genotoxicity, viability, and transcriptomic data generated for 18 individual MONPs, MPs, and their dissolved equivalents in a mouse lung epithelial cell in vitro system were used for a robust potency grouping and hazard identification exercise. The results showed that solubility in cell culture medium was clearly associated with the potency of MOs, with highly soluble MOs showing higher toxicity. The form of the metal oxide was also associated with loss in viable cell density and cell stress signaling (assessed through induction of the ‘HIF1α Signaling’ pathway), with dissolved metals showing higher potency overall for these endpoints, followed by NPs and MPs. The study shows that in vitro data can be used in a first-tier screening approach, to derive MONP potency groupings and identify hazards. Based on the results, CuO NPs, NiO NPs, and MnO_2_ NPs were identified as high-risk ENMs that require further testing using advanced toxicity models. For highly soluble ZnO, it is generally not necessary to assess the nanoform separately. However, it is important to consider the solubility of ZnO NPs in the exposure medium appropriate for all relevant routes of exposure. This ensures that any potential exposure to the particulate form is properly evaluated and not overlooked. For Al_2_O_3_, Fe_2_O_3_, and TiO_2_ NPs, the compounds were not identified as high risk in the short term, however firm conclusions could not be reached due to the potential for chronic toxicity resulting from exposure to these poorly soluble–insoluble MONPs. In summary, this study shows the potential of in vitro data in risk assessment processes, while drawing attention to its current limitations.

## Supplementary Information

Below is the link to the electronic supplementary material.

Additional methodological details and information pertaining to endpoint testing and BMC filtering (DOCX 50 kb)

Supplementary tables and figures pertaining to this manuscript (DOCX 885 kb)

List of genes in the ‘HIF1α Signaling’ IPA pathway corresponding to IPA content version 101138820, as well as concentration responding genes for each compound at 24 and 48 h (XLSX 19 kb)

Detailed description of compound grouping in the context of PCA analyses conducted at each BMR (DOCX 26 kb)

## Data Availability

Data is available upon request.
