# Supplementary material for: Applicability of in vitro mouse lung epithelial cell responses for potency grouping and hazard identification of metal oxide nanoparticles: impact of form, size, surface area, and solubility on toxicity
Source: Arch Toxicol. 2026 Jan 28;100(5):1995–2008. doi: 10.1007/s00204-025-04285-9 (PMC13086696; doi:10.1007/s00204-025-04285-9)
Supplement: Supplementary file 1 — Additional methodological details and information pertaining to endpoint testing and BMC filtering (DOCX 50 kb) [file 204_2025_4285_MOESM1_ESM.docx]

**Applicability of in vitro mouse lung epithelial cell response for potency grouping and hazard identification of metal oxide nanoparticles: Impact of form, size, surface area, and solubility on toxicity**

Andrey Boyadzhiev^1,2^, Andrew Williams^2^, Sabina Halappanavar^1,2,*^

1. Faculty of Science, University of Ottawa, Ottawa, Ontario, Canada

2. Environmental Health Science and Research Bureau, Health Canada, Ottawa, ON Canada.

* Corresponding author: [sabina.halappanavar@hc-sc.gc.ca](mailto:sabina.halappanavar@hc-sc.gc.ca)

## *Archives of Toxicology*

## ***In vitro* cell model used, cell exposure and endpoint analysis**

The details of cellular exposure, sample collection and the analysis of data from various endpoints, including the microarray gene expression data have been published previously (Boyadzhiev et al. 2021; Boyadzhiev et al. 2023; Christ 2024; Solorio-Rodriguez et al. 2024). Immortalized FE1 cells derived from the Muta™ Mouse transgenic rodent model were utilized for all exposures. These cells retain the characteristics of type I and type II pulmonary alveolar epithelial cells, and have been used in the past to assess the genotoxicity and mutagenicity of both chemicals and NPs (Arlt et al. 2008; Bengtson et al. 2016; Decan et al. 2016; Jacobsen et al. 2008; Maertens et al. 2017; White et al. 2003).

## **Viability endpoint data**

Viability endpoint data used for concentration modelling (viable cells / cm^2^) was measured through Trypan Blue exclusion staining after 24 and 48 h of exposure to 18 MONPs, MPs, and dissolved equivalents. Briefly, 10 µL of cell suspension was mixed with 10 µL of Trypan Blue dye. The suspension was allowed to incubate at room temperature for 5 – 10 min. After, 10 µL of the cell-dye suspension was loaded into a hemocytometer and the number of white and blue cells were counted per quadrant. At least three independent experiments were conducted with two technical replicates per condition.

## **Alkaline comet endpoint data**

The Trevigen’s 96-well CometChip^®^ system (Cedarlane Laboratories, Burlington, ON, Canada) was used to expose cells for 2 or 4 h, electrophorese, and fluorescently measure DNA damage (Boyadzhiev et al. 2022; Solorio-Rodriguez et al. 2024). For the negative control, cells were incubated in the cell culture medium for 4 h, and for the positive control, cells were exposed for 1 h to 100 μM H_2_O_2_ (Cat#216763, Sigma–Aldrich, Oakville, ON, Canada).

TIFF Images were generated for each gel using a Leica DMI8 automated confocal fluorescence microscope (Leica Microsystems, Wetzlar, Germany) at 5x magnification and were analyzed using the Trevigen Comet Software 1.3d (Bio-Techne, Devens, MA, USA). Artifacts were excluded from each well (double comet, debris, etc.). Wells with more than 50 comets were included in the final analysis, except for cells treated with high concentrations (50 µg/mL or 100 µg/mL) of CuO NPs, MnO_2_ NPs, NiO NPs, Fe_2_O_3_ NPs, MnO_2_ MPs, and Fe_2_O_3_ MPs, which resulted in non-uniform or incomplete staining of comet heads. This resulted in less than 50 comets/well. Thus, for high-concentration exposures, a decision was made to count the wells with less than 50 comets. Each experiment considered a minimum of 2 and a maximum of 8 technical replicates, with at least three independent experiments performed. The mean percentage of DNA in the tail was used as the metric for DNA strand breaks.

## ***In vitro* MicroFlow^®^ MN Assay**

Cells were exposed to varying concentrations of MONPs, MOMPs, or dissolved metal analogs for 40 h (Solorio-Rodriguez et al. 2024). According to the MicroFlow MN assay, cells should be exposed for a duration that approximates 1.5 to 2 normal cell cycles to evaluate MN induction. The doubling time of FE1 cells is 17 h; therefore, 40 h of exposures reflected cell division over 2 normal cell cycles. For the negative control, cells were incubated for 40 h in blank cell culture medium, whereas for the positive control, cells were exposed to 500 µM of methyl methanesulfonate (MMS) (Cat# 129925, Sigma–Aldrich, Oakville, ON, Canada) for 40 h. The MicroFlow kit (In Vitro MicroFlow kit, 1000/200, 96 well; Litron Laboratories, Rochester, NY, USA) was used to measure cytotoxicity, fold increase of apoptotic/necrotic cells, and MN frequency using flow cytometry. A Becton–Dickinson LSRFortessa^TM^ 5 laser analyzer (Becton–Dickinson, San Jose, CA, USA) was used to collect data. An analysis stop gate of 5000 ethidium monoazide (EMA)-negative nuclei was applied. The % relative survival, the increase of apoptotic/necrotic cells (EMA fold increase), and the MN frequency (% MN) were determined (Fortin et al. 2023).

% MN was scored in exposed cells and negative control using the double staining procedure outlined in the instruction manual. % MN was calculated as % MN = (MN Events/Nucleated Events) x 100. The final data represents 3–4 biological replicates per condition.

## **Microarray hybridization and statistical analysis**

The microarray samples were hybridized as described in (Decan et al. 2016). Statistical analysis and normalization (LOWESS method (locally weighted scatterplot smoothing)) of microarray data were conducted as described in (Poulsen et al. 2015). The log-normalized fluorescence ratios for each sample and biological replicate were used, the Agilent probe IDs were annotated according to Gene Symbol and then multiple transcript variants were collapsed down to one gene, taking the median response at each concentration for genes with more than one transcript variant. The microarray datasets used in this manuscript can be found in the NCBI gene expression omnibus, under the accession number GSE246159 and GSE161017.

## **BMC modelling of transcriptomic data**

The transcriptomic BMC data was truncated to 198 genes belonging to the ‘HIF1α Signaling’ pathway. This single pathway was chosen due to 1) its consistent expression across the tested MONPs, 2) its strong correlation with *in vitro* cytotoxicity, and 3) due to its association with stress responses relevant to the toxicity of MONPs (Boyadzhiev et al. 2023).

**BMC filtering and the final matrices used for downstream analysis**

The initial BMC matrices were filtered to remove extreme outlier values resulting from imprecise BMC estimation. At BMR5 and BMR10, both 24 h viability BMC and MN BMC values were removed for MnO_2_ NPs (8 x 10^12^ – 2.7 x 10^-6^ µM metal). At BMR25, 24 h viability BMC for Al_2_O_3_ MPs and MnO_2_ NPs (1.4 x 10^5^, 8 x 10^13^ µM metal respectively), as well as 48 h viability BMC for Al_2_O_3_ MPs (1.4 x 10^5^ µM metal), and MN BMC for MnO_2_ NPs (3.4 x 10^-5^ µM metal) were removed. Finally at BMR50, three values were removed, including 24 h viability BMC for MnO_2_ NPs and Al_2_O_3_ MPs (2 x 10^14^, 2.4 x 10^9^ µM metal respectively) and 48 h viability BMC for Al_2_O_3_ MPs (2.4 x 10^9^ µM metal). No additional filtering was performed in order to keep as many original BMC values as possible for grouping purposes. The resulting BMC matrices were Winsorized in R to the 5^th^ / 95^th^ percentile, log_10_ transformed, scaled, and used for downstream analyses. Each starting matrix consisted of 104 computed BMC values expressed in µM of the constituent metal so that all MONP forms could be assessed using a relevant concentration metric. After filtering and Winsorization to account for extreme outlier values resulting from imprecise BMC calculation, 88 calculated BMCs for BMR5 – 10, 86 calculated BMCs for BMR25, and 87 calculated BMCs at BMR50 were retained (Supplementary Tables 3. – 6.).

Across the apical endpoints, 48 h viability was the most robust endpoint for modelling across all BMRs, with 1 BMC replaced with the lower 5^th^ percentile, 2 BMCs replaced with the upper 95^th^ percentile at BMR5 – 25, and 3 BMCs replaced with the upper 95^th^ percentile at BMR50.

In contrast, 24 h ‘HIF1α Signaling’ tPOD was the least robust, with 7/18 compounds not having a BMC and replaced with the upper 95^th^ percentile and 1 calculated BMC replaced with the lower 5^th^ percentile at each BMR.

**Correlation analyses between endpoints and physicochemical properties**

In order to determine relationships between endpoints and key physicochemical properties, the Kendall’s Tau-b was used. This correlation test is specifically suited for handling ties in the data (Kendall 1945), which are present when all BMC data across BMRs is combined with compound-specific physicochemical properties. For this purpose, 3 separate correlations were conducted. In the first, all samples were used (n = 72) along with a property called ‘form’. This property defines whether the compound was a dissolved metal (form = 1), a nanoparticle (form = 2), or a microparticle (form = 3). In the second analysis, all dissolved metals were removed and PPS and SSA were included as physicochemical properties (n = 56). In the final comparison, all dissolved metals in addition to Fe_2_O_3_ NPs and MPs were removed and solubility at 100 µg/mL was included as a property (n = 48). Fe_2_O_3_ particles were not included as there was no quantifiable dissolution measured for these materials past the 0 h timepoint (Avramescu et al. 2022). An association was considered significant if the p < 0.05. For simplicity, the strength of association was judged the same as for the Spearman’s coefficient.

**References**

Arlt VM, Gingerich J, Schmeiser HH, Phillips DH, Douglas GR, White PA (2008) Genotoxicity of 3-nitrobenzanthrone and 3-aminobenzanthrone in MutaMouse and lung epithelial cells derived from MutaMouse. Mutagenesis 23:483. doi: 10.1093/mutage/gen037

Avramescu M, Chénier M, Beauchemin S, Rasmussen P (2022) Dissolution Behaviour of Metal-Oxide Nanomaterials in Various Biological Media. Nanomaterials (Basel, Switzerland) 13:26. doi: 10.3390/nano13010026

Bengtson S, Kling K, Madsen AM, Noergaard AW, Jacobsen NR, Clausen PA, Alonso B, Pesquera A, Zurutuza A, Ramos R, Okuno H, Dijon J, Wallin H, Vogel U (2016) No cytotoxicity or genotoxicity of graphene and graphene oxide in murine lung epithelial FE1 cells in vitro. Environmental and molecular mutagenesis 57:469–482. doi: 10.1002/em.22017

Boyadzhiev A, Avramescu M, Wu D, Williams A, Rasmussen P, Halappanavar S (2021) Impact of copper oxide particle dissolution on lung epithelial cell toxicity: response characterization using global transcriptional analysis. Nanotoxicology 15:380–399. doi: 10.1080/17435390.2021.1872114

Boyadzhiev A, Solorio-Rodriguez SA, Wu D, Avramescu M, Rasmussen P, Halappanavar S (2022) The High-Throughput In Vitro CometChip Assay for the Analysis of Metal Oxide Nanomaterial Induced DNA Damage. Nanomaterials (Basel, Switzerland) 12:1844. doi: 10.3390/nano12111844

Boyadzhiev A, Wu D, Avramescu M, Williams A, Rasmussen P, Halappanavar S (2023) Toxicity of Metal Oxide Nanoparticles: Looking through the Lens of Toxicogenomics. International journal of molecular sciences 25:529. doi: 10.3390/ijms25010529

Christ C (2024) Establishing the impact of metal oxide nanoparticle size and
solubility on lung epithelial cell toxicity. Masters of Science, University of Ottawa

Decan N, Wu D, Williams A, Bernatchez S, Johnston M, Hill M, Halappanavar S (2016) Characterization of in vitro genotoxic, cytotoxic and transcriptomic responses following exposures to amorphous silica of different sizes. Mutation research. Genetic toxicology and environmental mutagenesis 796:8–22. doi: 10.1016/j.mrgentox.2015.11.011

Fortin AV, Long AS, Williams A, Meier MJ, Cox J, Pinsonnault C, Yauk CL, White PA (2023) Application of a new approach methodology (NAM)-based strategy for genotoxicity assessment of data-poor compounds. Frontiers in toxicology 5:1098432. doi: 10.3389/ftox.2023.1098432

Jacobsen NR, Pojana G, White P, Møller P, Cohn CA, Smith Korsholm K, Vogel U, Marcomini A, Loft S, Wallin H (2008) Genotoxicity, cytotoxicity, and reactive oxygen species induced by single-walled carbon nanotubes and C₆₀ fullerenes in the FE1-Muta[trade mark sign]Mouse lung epithelial cells. Environmental and Molecular Mutagenesis 49:476–487. doi: 10.1002/em.20406

Kendall MG (1945) THE TREATMENT OF TIES IN RANKING PROBLEMS. Biometrika 33:239–251. doi: 10.1093/biomet/33.3.239

Maertens RM, Long AS, White PA, Benthem Jv (2017) Performance of the in vitro transgene mutation assay in MutaMouse FE1 cells: Evaluation of nine misleading (“False”) positive chemicals. Environmental and molecular mutagenesis 58:582–591. doi: 10.1002/em.22125

Poulsen SS, Saber AT, Williams A, Andersen O, Købler C, Atluri R, Pozzebon ME, Mucelli SP, Simion M, Rickerby D, Mortensen A, Jackson P, Kyjovska ZO, Mølhave K, Jacobsen NR, Jensen KA, Yauk CL, Wallin H, Halappanavar S, Vogel U (2015) MWCNTs of different physicochemical properties cause similar inflammatory responses, but differences in transcriptional and histological markers of fibrosis in mouse lungs. Toxicology and Applied Pharmacology 284:16–32. doi: 10.1016/j.taap.2014.12.011

Solorio-Rodriguez SA, Wu D, Boyadzhiev A, Christ C, Williams A, Halappanavar S (2024) A Systematic Genotoxicity Assessment of a Suite of Metal Oxide Nanoparticles Reveals Their DNA Damaging and Clastogenic Potential. Nanomaterials (Basel, Switzerland) 14:743. doi: 10.3390/nano14090743

White PA, Douglas GR, Gingerich J, Parfett C, Shwed P, Seligy V, Soper L, Berndt L, Bayley J, Wagner S, Pound K, Blakey D (2003) Development and characterization of a stable epithelial cell line from Muta™Mouse lung. Environmental and molecular mutagenesis 42:166–184. doi: 10.1002/em.10185
