# Supplementary material for: Applicability of in vitro mouse lung epithelial cell responses for potency grouping and hazard identification of metal oxide nanoparticles: impact of form, size, surface area, and solubility on toxicity
Source: Arch Toxicol. 2026 Jan 28;100(5):1995–2008. doi: 10.1007/s00204-025-04285-9 (PMC13086696; doi:10.1007/s00204-025-04285-9)
Supplement: Supplementary file 2 — Supplementary tables and figures pertaining to this manuscript (DOCX 885 kb) [file 204_2025_4285_MOESM2_ESM.docx]

**Applicability of in vitro mouse lung epithelial cell response for potency grouping and hazard identification of metal oxide nanoparticles: Impact of form, size, surface area, and solubility on toxicity**

Andrey Boyadzhiev^1,2^, Andrew Williams^2^, Sabina Halappanavar^1,2,*^

1. Faculty of Science, University of Ottawa, Ottawa, Ontario, Canada

2. Environmental Health Science and Research Bureau, Health Canada, Ottawa, ON Canada.

* Corresponding author: [sabina.halappanavar@hc-sc.gc.ca](mailto:sabina.halappanavar@hc-sc.gc.ca)

## *Archives of Toxicology*

**Supplementary Table. 1** Filtered and Winsorized benchmark dose (BMC) matrix at a benchmark response (BMR) of 5% relative risk. All concentrations are in µM of the constituent metal. NP: nanoparticle. MP: microparticle. tPOD: transcriptional point of departure. Red: value replaced with upper 95^th^ percentile. Green: value replaced with lower 5^th^ percentile.

| **Compounds** | **24 h Viability BMC** | **48 h Viability BMC** | **24 h Hif1a (Median)** | **48 h Hif1a (Median)** | **40 h**  **MN BMC** | **2 h Comet BMC** | **4 h Comet BMC** |
| --- | --- | --- | --- | --- | --- | --- | --- |
| ZnCl_2_ | 32.11 | 28.42 | 33.91 | 36.11 | 4.74E-05 | 88.70 | 29.35 |
| ZnO NP | 9.87 | 8.60 | 23.05 | 21.82 | 3.61E-05 | 106.76 | 31.14 |
| ZnO MP | 16.45 | 11.28 | 20.21 | 20.87 | 0.02 | 135.50 | 52.38 |
| CuO NP | 48.54 | 24.63 | 126.39 | 44.69 | 0.11 | 9.79 | 3.91 |
| CuO MP | 515.78 | 505.38 | 505.58 | 397.91 | 7.48 | 124.36 | 217.48 |
| MnSO_4_ | 9.37 | 9.37 | 16.41 | 30.92 | 60.26 | 135.50 | 217.48 |
| MnO_2_ NP | 515.78 | 58.76 | 222.58 | 355.27 | 3.61E-05 | 26.13 | 16.00 |
| MnO_2_ MP | 463.79 | 463.79 | 239.94 | 222.40 | 4.63 | 86.47 | 48.07 |
| NiCl_2_ | 59.07 | 30.19 | 58.97 | 75.44 | 15.34 | 135.50 | 217.48 |
| NiO NP | 57.90 | 13.43 | 159.77 | 227.84 | 0.02 | 20.82 | 6.32 |
| NiO MP | 117.57 | 117.57 | 141.26 | 163.02 | 0.02 | 6.98 | 4.45 |
| AlCl_3_ | 224.54 | 26.85 | 505.58 | 129.94 | 0.00 | 135.50 | 217.48 |
| Al_2_O_3_ NP | 91.35 | 91.35 | 505.58 | 206.91 | 25.48 | 7.97 | 176.38 |
| Al_2_O_3_ MP | 4.47 | 4.77 | 505.58 | 397.91 | 60.26 | 135.50 | 217.48 |
| Fe_2_O_3_ NP | 327.63 | 327.63 | 505.58 | 243.24 | 60.26 | 34.45 | 34.45 |
| Fe_2_O_3_ MP | 5.95 | 5.95 | 505.58 | 382.21 | 2.27 | 19.88 | 19.88 |
| TiO_2_ NP | 140.36 | 66.21 | 502.19 | 165.55 | 60.26 | 10.86 | 10.86 |
| TiO_2_ MP | 515.78 | 505.38 | 505.58 | 397.91 | 60.26 | 5.04 | 40.74 |

**Supplementary Table. 2** Filtered and Winsorized benchmark concentration (BMC) matrix at a benchmark response (BMR) of 10% relative risk. All concentrations are in µM of the constituent metal. NP: nanoparticle. MP: microparticle. tPOD: transcriptional point of departure. Red: value replaced with upper 95^th^ percentile. Green: value replaced with lower 5^th^ percentile.

| **Compounds** | **24 h Viability BMC** | **48 h Viability BMC** | **24 h Hif1α (Median)** | **48 h Hif1α (Median)** | **40 h**  **MN BMC** | **2 h Comet BMC** | **4 h Comet BMC** |
| --- | --- | --- | --- | --- | --- | --- | --- |
| ZnCl_2_ | 37.08 | 32.82 | 37.35 | 38.24 | 2.72E-03 | 195.38 | 65.71 |
| ZnO NP | 16.12 | 14.09 | 27.24 | 26.72 | 3.15E-03 | 140.08 | 40.86 |
| ZnO MP | 34.65 | 29.76 | 23.93 | 25.46 | 0.18 | 196.61 | 65.96 |
| CuO NP | 62.71 | 31.82 | 158.61 | 55.14 | 0.30 | 19.39 | 7.00 |
| CuO MP | 765.77 | 752.67 | 632.38 | 500.17 | 10.70 | 203.92 | 513.92 |
| MnSO_4_ | 18.17 | 18.17 | 19.93 | 38.01 | 101.98 | 203.92 | 711.92 |
| MnO_2_ NP | 765.77 | 165.06 | 312.76 | 431.26 | 2.72E-03 | 23.11 | 14.47 |
| MnO_2_ MP | 700.28 | 700.28 | 296.70 | 277.32 | 8.40 | 157.93 | 87.78 |
| NiCl_2_ | 84.98 | 43.46 | 78.71 | 104.74 | 31.27 | 203.92 | 711.92 |
| NiO NP | 119.61 | 27.74 | 214.06 | 277.40 | 0.14 | 25.54 | 7.66 |
| NiO MP | 222.24 | 222.24 | 201.89 | 205.98 | 0.18 | 17.71 | 11.30 |
| AlCl_3_ | 427.04 | 143.27 | 632.38 | 171.42 | 0.03 | 203.92 | 711.92 |
| Al_2_O_3_ NP | 275.01 | 275.01 | 632.38 | 283.52 | 43.39 | 48.76 | 711.92 |
| Al_2_O_3_ MP | 15.61 | 14.09 | 632.38 | 500.17 | 101.98 | 203.92 | 711.92 |
| Fe_2_O_3_ NP | 500.47 | 500.47 | 632.38 | 328.69 | 101.98 | 146.03 | 146.03 |
| Fe_2_O_3_ MP | 47.38 | 47.38 | 632.38 | 476.99 | 7.37 | 57.62 | 57.62 |
| TiO_2_ NP | 238.77 | 112.64 | 627.09 | 259.15 | 101.98 | 83.75 | 83.75 |
| TiO_2_ MP | 765.77 | 752.67 | 632.38 | 500.17 | 101.98 | 16.03 | 367.49 |

**Supplementary Table. 3** Filtered and Winsorized benchmark concentration (BMC) matrix at a benchmark response (BMR) of 25% relative risk. All concentrations are in µM of the constituent metal. NP: nanoparticle. MP: microparticle. tPOD: transcriptional point of departure. Red: value replaced with upper 95^th^ percentile. Green: value replaced with lower 5^th^ percentile.

| **Compounds** | **24 h Viability BMC** | **48 h Viability BMC** | **24 h Hif1α (Median)** | **48 h Hif1α (Median)** | **40 h**  **MN BMC** | **2 h Comet BMC** | **4 h Comet BMC** |
| --- | --- | --- | --- | --- | --- | --- | --- |
| ZnCl_2_ | 45.36 | 40.15 | 43.04 | 44.58 | 0.11 | 286.87 | 96.48 |
| ZnO NP | 32.00 | 27.86 | 33.66 | 34.69 | 0.16 | 200.05 | 58.34 |
| ZnO MP | 31.09 | 25.86 | 29.38 | 32.94 | 3.22 | 266.16 | 89.30 |
| CuO NP | 94.90 | 48.16 | 207.45 | 69.75 | 1.26 | 47.14 | 14.07 |
| CuO MP | 1803.59 | 1437.79 | 860.06 | 671.95 | 17.29 | 487.15 | 1151.17 |
| MnSO_4_ | 45.89 | 45.89 | 25.47 | 48.51 | 201.35 | 989.27 | 7701.83 |
| MnO_2_ NP | 1803.59 | 698.78 | 485.09 | 532.66 | 0.11 | 114.92 | 70.36 |
| MnO_2_ MP | 1245.73 | 1245.73 | 390.78 | 368.44 | 29.44 | 376.37 | 209.23 |
| NiCl_2_ | 153.18 | 78.30 | 114.65 | 148.94 | 63.19 | 989.27 | 7701.83 |
| NiO NP | 360.14 | 83.52 | 309.97 | 355.29 | 1.76 | 40.10 | 11.83 |
| NiO MP | 541.15 | 541.15 | 320.05 | 278.55 | 2.41 | 37.97 | 21.70 |
| AlCl_3_ | 1803.59 | 497.04 | 860.06 | 244.84 | 0.61 | 989.27 | 7701.83 |
| Al_2_O_3_ NP | 1283.44 | 1283.44 | 860.06 | 425.76 | 89.21 | 488.23 | 7701.83 |
| Al_2_O_3_ MP | 1803.59 | 1437.79 | 860.06 | 671.95 | 201.35 | 989.27 | 7701.83 |
| Fe_2_O_3_ NP | 993.80 | 993.80 | 854.49 | 484.77 | 201.35 | 916.65 | 916.65 |
| Fe_2_O_3_ MP | 1361.39 | 1361.39 | 860.06 | 634.81 | 40.45 | 289.81 | 289.81 |
| TiO_2_ NP | 720.96 | 340.07 | 860.06 | 466.20 | 201.35 | 989.27 | 1124.13 |
| TiO_2_ MP | 1666.98 | 1437.79 | 860.06 | 671.95 | 201.35 | 211.53 | 6029.18 |

**Supplementary Table. 4** Filtered and Winsorized benchmark concentration (BMC) matrix at a benchmark response (BMR) of 50% relative risk. All concentrations are in µM of the constituent metal. NP: nanoparticle. MP: microparticle. tPOD: transcriptional point of departure. Red: value replaced with upper 95^th^ percentile. Green: value replaced with lower 5^th^ percentile.

| **Compounds** | **24 h Viability BMC** | **48 h Viability BMC** | **24 h Hif1α (Median)** | **48 h Hif1α (Median)** | **40 h**  **MN BMC** | **2 h Comet BMC** | **4 h Comet BMC** |
| --- | --- | --- | --- | --- | --- | --- | --- |
| ZnCl_2_ | 54.13 | 47.92 | 48.07 | 49.28 | 0.98 | 385.25 | 129.57 |
| ZnO NP | 58.53 | 50.96 | 40.12 | 42.68 | 1.36 | 261.12 | 76.17 |
| ZnO MP | 50.03 | 42.87 | 34.91 | 40.45 | 24.49 | 334.23 | 112.10 |
| CuO NP | 147.09 | 74.66 | 275.48 | 88.61 | 3.95 | 90.89 | 27.12 |
| CuO MP | 22772.68 | 16504.43 | 1113.42 | 857.74 | 25.36 | 932.30 | 2202.53 |
| MnSO_4_ | 103.54 | 103.54 | 32.40 | 58.07 | 311.43 | 4614.49 | 47051.25 |
| MnO_2_ NP | 22772.68 | 2483.41 | 692.34 | 653.68 | 0.64 | 232.35 | 142.17 |
| MnO_2_ MP | 2065.87 | 2065.87 | 491.25 | 497.28 | 70.89 | 757.22 | 421.00 |
| NiCl_2_ | 285.62 | 145.99 | 159.36 | 177.13 | 111.24 | 4614.49 | 47051.25 |
| NiO NP | 1057.40 | 245.27 | 419.36 | 436.57 | 10.63 | 73.01 | 25.38 |
| NiO MP | 1182.71 | 1182.71 | 461.78 | 357.47 | 15.24 | 69.34 | 39.88 |
| AlCl_3_ | 12173.30 | 2850.52 | 1113.42 | 328.93 | 6.64 | 4614.49 | 47051.25 |
| Al_2_O_3_ NP | 6171.05 | 6171.05 | 1113.42 | 595.43 | 161.10 | 2467.63 | 47051.25 |
| Al_2_O_3_ MP | 22772.68 | 16504.43 | 1113.42 | 857.74 | 311.43 | 4614.49 | 47051.25 |
| Fe_2_O_3_ NP | 2052.73 | 2052.73 | 1087.26 | 668.47 | 311.43 | 3336.47 | 3336.47 |
| Fe_2_O_3_ MP | 22772.68 | 16504.43 | 1113.42 | 803.97 | 159.18 | 832.36 | 832.36 |
| TiO_2_ NP | 2712.04 | 5747.13 | 1113.42 | 743.88 | 311.43 | 4614.49 | 6987.95 |
| TiO_2_ MP | 3019.60 | 3019.60 | 1113.42 | 857.74 | 311.43 | 1512.93 | 42970.77 |


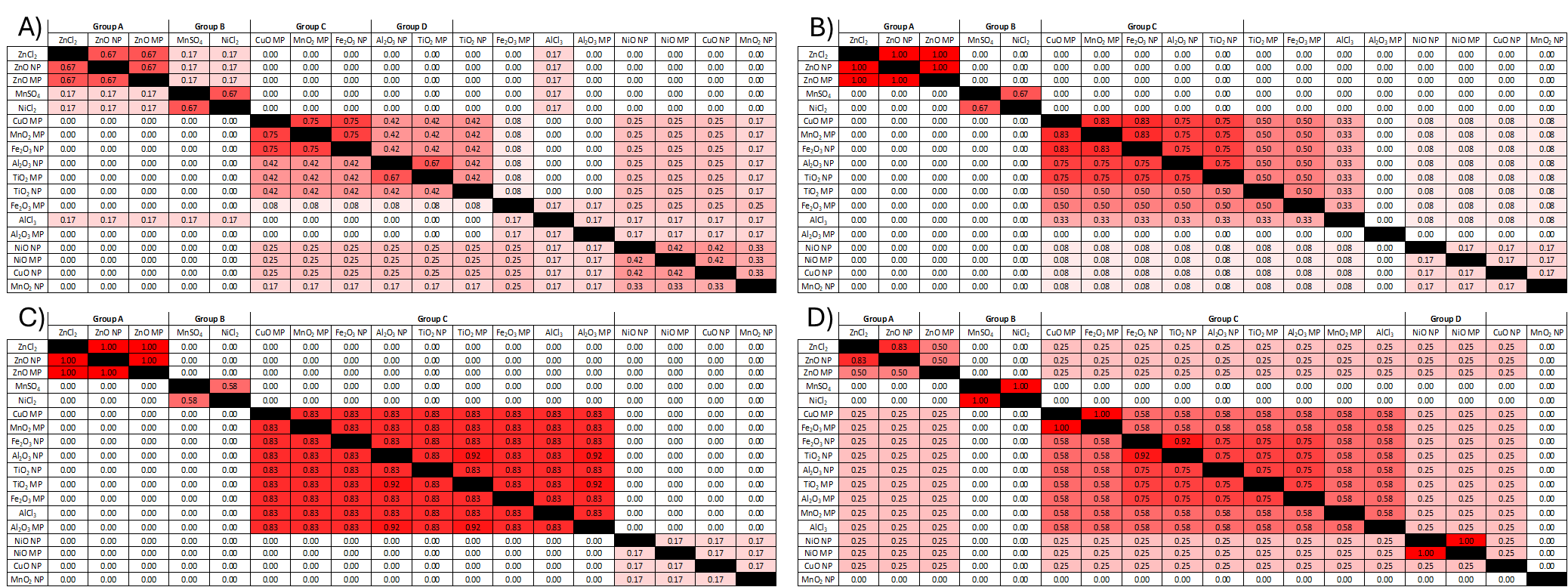


**Supplementary Fig. 1** Jaccard similarity measures showing the likelihood of MONPs, MOMPs, and dissolved equivalents to appear in the same statistically supported hierarchical cluster based on potency across *in vitro* endpoints at a BMR of A) 5%, B) 10%, C) 25%, or D) 50%. Each matrix was constructed based on 12 hierarchical clustering attempts. Compounds were considered to group together if they appeared in the same statistically supported cluster >50% of the time. 1: substances always appear in the same supported cluster. 0: substances never appear in the same supported cluster. Red: higher likelihood of appearing in the same supported cluster. Black: non-applicable comparison. NP: nanoparticle. MP: microparticle.

**Supplementary Table. 5** Variance and loading analysis corresponding to PCA analysis of BMR5, 10, 25, and 50% matrices. PC: principle component. Numbers in bold indicate significant loading onto a PC.

|  | | PC1 (BMR5) | PC2 (BMR5) | PC1 (BMR10) | PC2 (BMR10) | PC1 (BMR25) | PC2 (BMR25) | PC1 (BMR50) | PC2 (BMR50) |
| --- | --- | --- | --- | --- | --- | --- | --- | --- | --- |
| Variance | Standard Deviation | 1.80 | 1.29 | 1.87 | 1.38 | 2.09 | 1.38 | 2.13 | 1.35 |
|  | Proportion of Variance | 0.46 | 0.24 | 0.50 | 0.27 | 0.63 | 0.27 | 0.65 | 0.26 |
|  | Cumulative Proportion | 0.46 | 0.70 | 0.50 | 0.77 | 0.63 | 0.90 | 0.65 | 0.91 |
| Loadings | 24 h Viability | **0.43** | -0.08 | **0.46** | -0.16 | **0.45** | -0.23 | **0.42** | 0.29 |
|  | 48 h Viability | **0.44** | 0.01 | **0.46** | -0.03 | **0.45** | -0.16 | **0.44** | 0.19 |
|  | 24 h 'HIF1α Signaling' tPOD | **0.48** | 0.12 | **0.48** | -0.01 | **0.43** | -0.25 | **0.42** | 0.30 |
|  | 48 h 'HIF1α Signaling' tPOD | **0.47** | 0.13 | **0.48** | -0.01 | **0.44** | -0.24 | **0.43** | 0.27 |
|  | 40 h Micronucleus | 0.26 | **0.44** | 0.26 | **0.46** | 0.31 | 0.38 | 0.30 | **-0.41** |
|  | 2 h Comet | -0.33 | **0.48** | -0.17 | **0.57** | 0.20 | **0.61** | 0.29 | **-0.55** |
|  | 4 h Comet | -0.06 | **0.73** | 0.12 | **0.66** | 0.28 | **0.54** | 0.31 | **-0.51** |


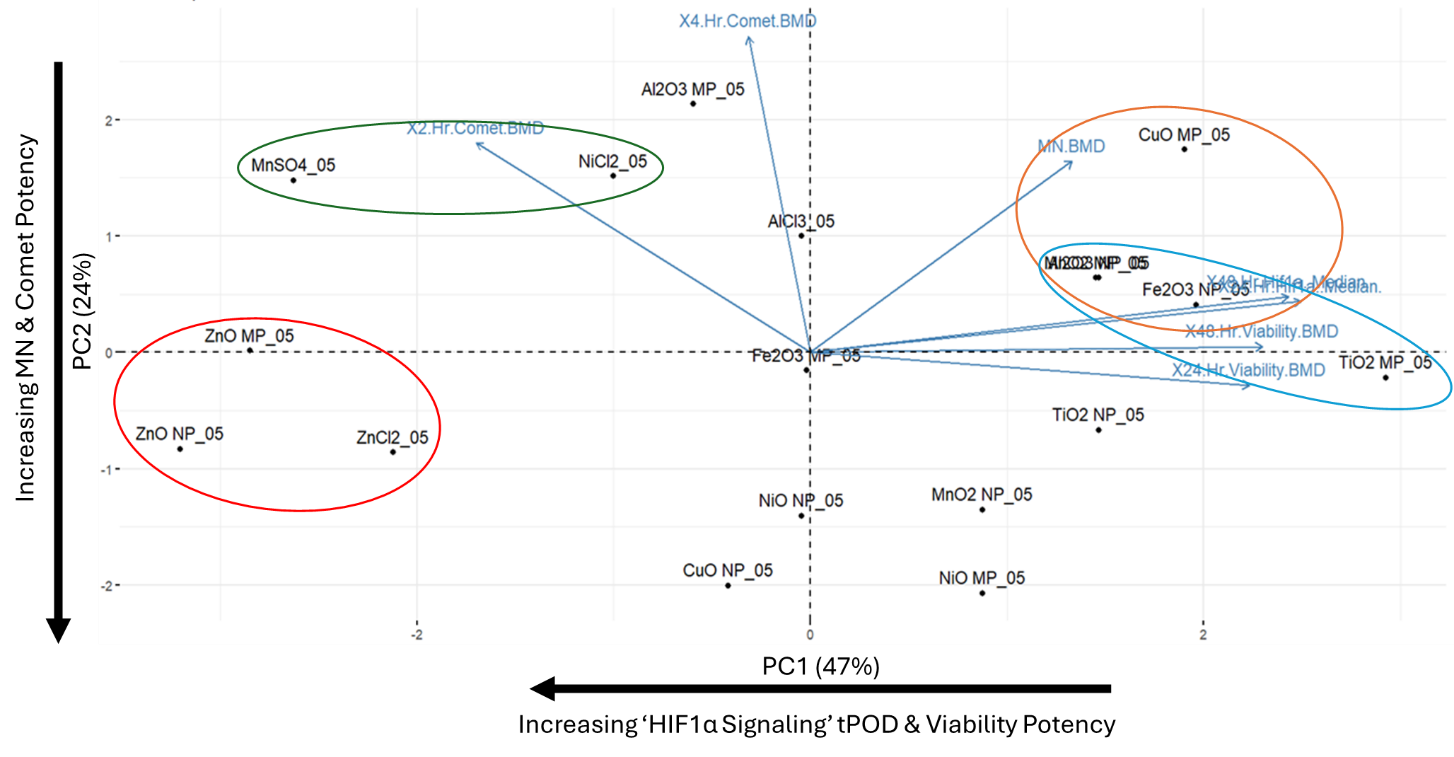


**Supplementary Fig. 2** Biplot showing principle component (PC1) and PC2 from (principle component analysis) (PCA) of filtered, log transformed, and scaled BMR05 BMC matrix. Vectors indicate the direction of loading for each endpoint. Viability, and ‘HIF1α Signaling’ tPOD endpoints load significantly and positively onto PC1. Comet and micronucleus (MN) endpoints load significantly and positively onto PC2. Numbers in parentheses indicate the amount of variance explained by each PC. Colored circles indicate compounds likely to cluster together based on 12 hierarchical clustering attempts.


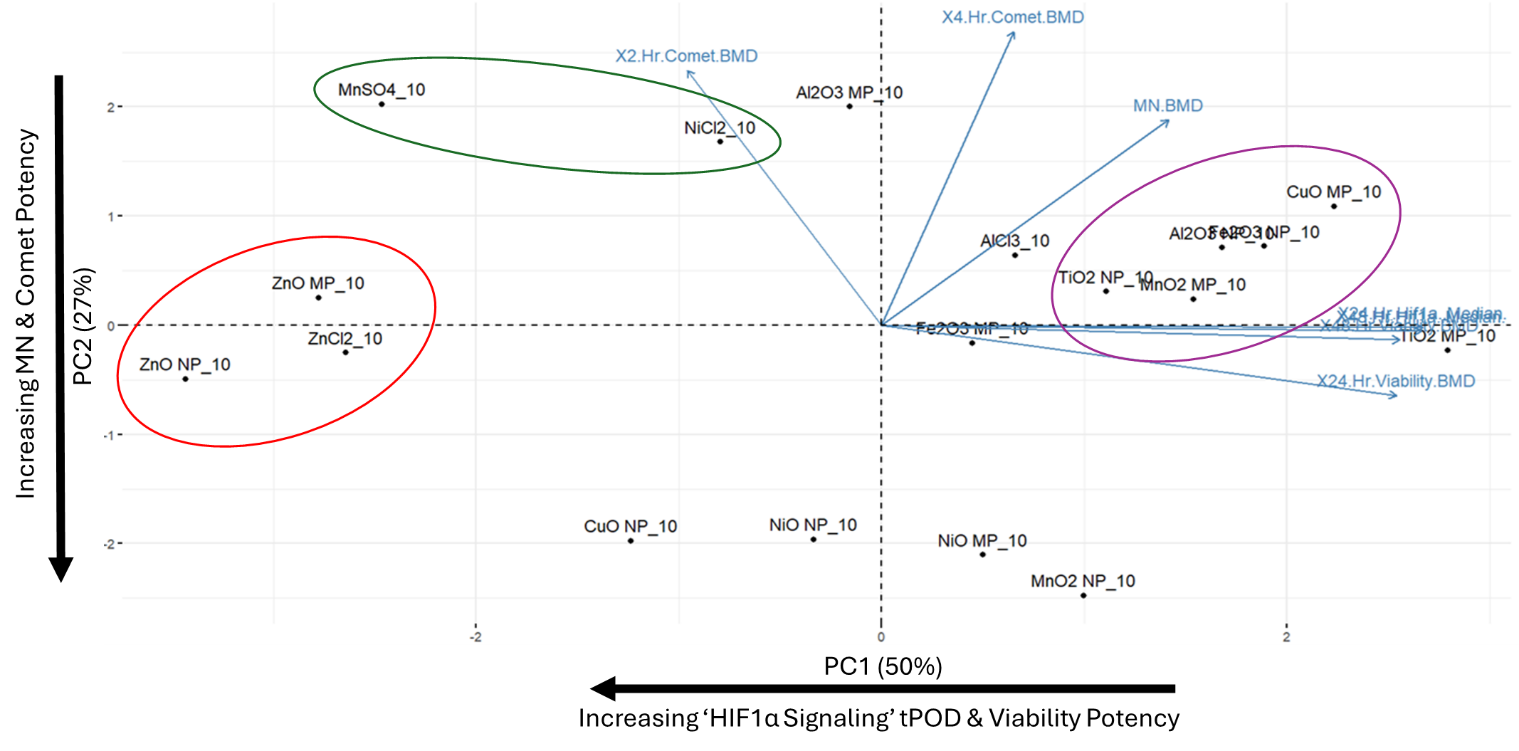


**Supplementary Fig. 3** Biplot showing principle component (PC1) and PC2 from (principle component analysis) (PCA) of filtered, log transformed, and scaled BMR10 BMC matrix. Vectors indicate the direction of loading for each endpoint. Viability and ‘HIF1α Signaling’ endpoints load significantly and positively onto PC1. Comet and MN endpoints load significantly and positively onto PC2. Numbers in parentheses indicate the amount of variance explained by each PC. Colored circles indicate compounds likely to cluster together based on 12 hierarchical clustering attempts.


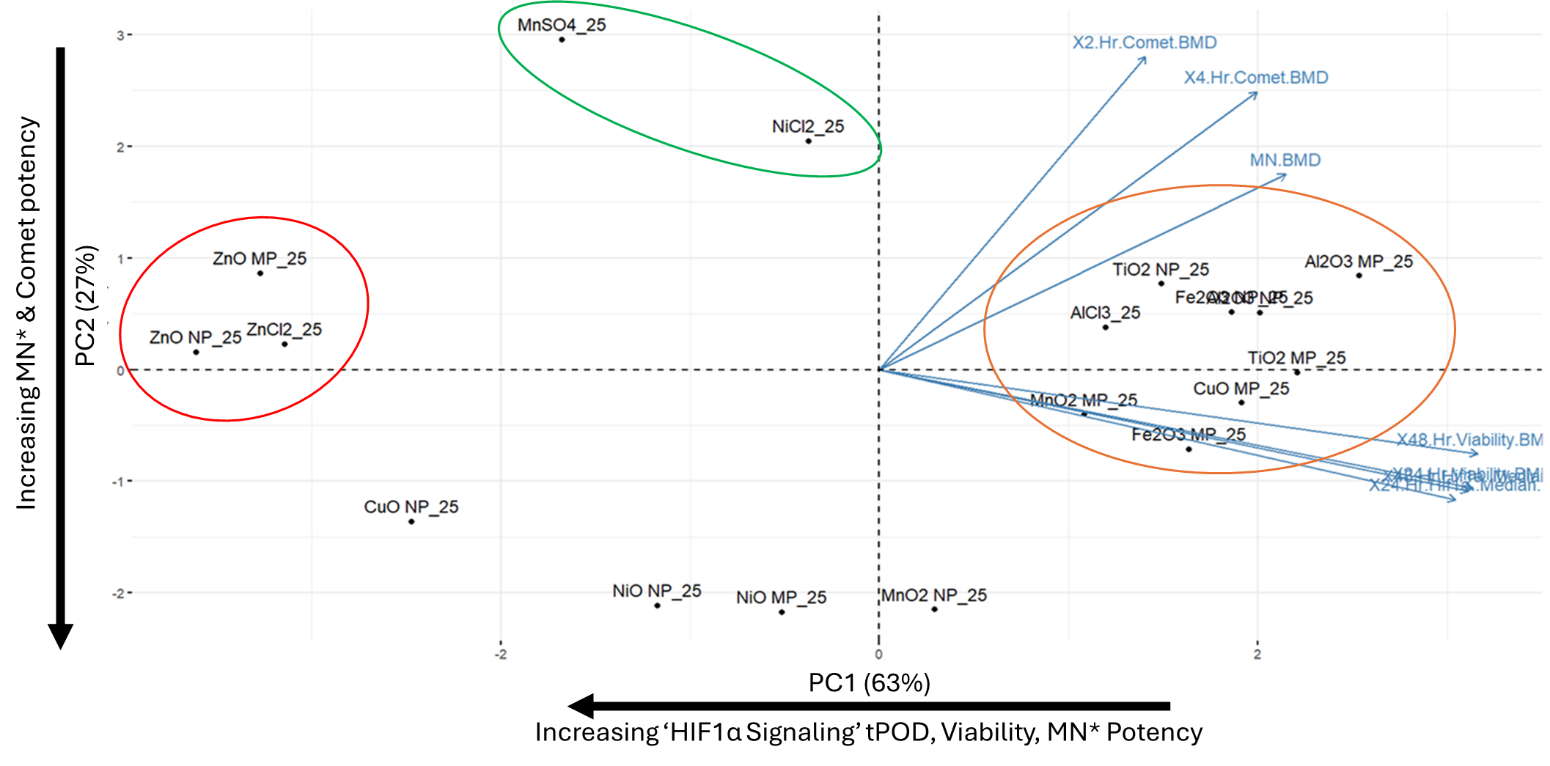


**Supplementary Fig. 4** Biplot showing principle component (PC1) and PC2 from (principle component analysis) (PCA) of filtered, log transformed, and scaled BMR25 BMC matrix. Vectors indicate the direction of loading for each endpoint. Viability, and ‘HIF1α Signaling’ endpoints load significantly and positively onto PC1. Comet endpoints load significantly and positively onto PC2. The micronucleus (MN*) endpoint loads in the same direction as all endpoints and both PCs, but less than the 0.4 cutoff. Numbers in parentheses indicate the amount of variance explained by each PC. Colored circles indicate compounds likely to cluster together based on 12 hierarchical clustering attempts.
