# Supplementary material for: Applicability of in vitro mouse lung epithelial cell responses for potency grouping and hazard identification of metal oxide nanoparticles: impact of form, size, surface area, and solubility on toxicity
Source: Arch Toxicol. 2026 Jan 28;100(5):1995–2008. doi: 10.1007/s00204-025-04285-9 (PMC13086696; doi:10.1007/s00204-025-04285-9)
Supplement: Supplementary file 4 — Detailed description of compound grouping in the context of PCA analyses conducted at each BMR (DOCX 26 kb) [file 204_2025_4285_MOESM4_ESM.docx]

**Applicability of in vitro mouse lung epithelial cell response for potency grouping and hazard identification of metal oxide nanoparticles: Impact of form, size, surface area, and solubility on toxicity**

Andrey Boyadzhiev^1,2^, Andrew Williams^2^, Sabina Halappanavar^1,2,*^

1. Faculty of Science, University of Ottawa, Ottawa, Ontario, Canada

2. Environmental Health Science and Research Bureau, Health Canada, Ottawa, ON Canada.

* Corresponding author: [sabina.halappanavar@hc-sc.gc.ca](mailto:sabina.halappanavar@hc-sc.gc.ca)

## *Archives of Toxicology*

## **Compound groupings as they appear one PC1-PC2 biplots across BMR5-50:**

Compound groupings identified through hierarchical clustering were used to annotate the PC1 – PC2 biplots, which represent cell death / cell stress and genotoxicity respectively.

Zn compounds which appear as part of Group ‘A’ have highly negative values along PC1 and values around -1 to +1 along PC2 at all BMRs (Fig. 2. Online Resource 2; Supplementary Fig. 2 – 4). This indicates a moderate genotoxic potential, but a very strong potency to induce cell stress and cell death regardless of the BMR chosen.

The consistent Group ‘B’ made up of NiCl_2_ and MnSO_4_ shows change in position on the PC1 and PC2 biplots across BMRs. At BMR5, both NiCl_2_ and MnSO_4_ have similar values along the PC2 reflecting genotoxicity potential (Online Resource 2; Supplementary Fig. 2), while MnSO_4_ has much more negative values along PC1 as compared to NiCl_2_. These results suggest a similar genotoxic potential, but stronger potency to induce cell stress and cell death from MnSO_4_ as compared to NiCl_2_. As the BMR increases, MnSO_4_ becomes more separated along PC2 from NiCl_2_, indicating a progressively weaker potency to induce genotoxicity as compared to NiCl_2_. Furthermore, the relative potency to induce cell death and cell stress decreases as the BMRs increase, with MnSO_4_ presenting similar relative potency along PC1 as Zn compounds at BMR5, whereas at BMR50 the position is similar to NiO NPs.

For the 9 compounds which make up Group ‘C’ at BMR25-50 (Fig. 2. Online Resource 2; Supplementary Fig. 4.), they appear progressively closer together as BMR increases (Fig. 2. Online Resource 2; Supplementary Fig. 3 – 4), with a range of negative values along PC1 and values along PC2 between +1 and -1. Together this indicates a weak potential to induce cell stress and cell death, and a moderate but variable potency to induce genotoxicity.

For NiO NPs and MPs, which form Group ‘D’ at BMR50, it can be seen that they are in close proximity along PC1 and PC2 at all BMRs, but the distance between them becomes less as the BMR increases. Across all BMRs, NiO NPs present more negative values along PC1 than NiO MPs, indicating a relatively greater potential to induce cell death and cell stress from NPs as compared to MPs. For NiO NPs and MPs, they always present high values along PC2 indicative of strong genotoxic potency relative to the other 16 compounds tested.

For MnO_2_ NPs and CuO NPs which do not frequently cluster with any compound, they can both be seen in close proximity to NiO NPs and MPs at BMR5 (Online Resource 2; Supplementary Fig. 2). However, while MnO_2_ NPs stay in relative proximity to NiO particles as BMRs increase, CuO NPs move progressively towards Zn compounds presenting higher potential to induce cell stress and cell death at higher BMRs (Fig. 2. Online Resource 2; Supplementary Fig. 3-4). Both MnO_2_ NPs and CuO NPs exhibit pronounced genotoxic potential based on their position along PC2, however CuO NPs have more negative values along PC1 whose magnitude increases as BMR increases. This indicates strong genotoxic potential for both CuO NPs and MnO_2_ NPs, but a stronger potency to induce cell stress and cell death in a BMR-dependent manner.
